# MdMYB10 affects nitrogen uptake and reallocation by regulating the nitrate transporter MdNRT2.4-1 in red-fleshed apple

**DOI:** 10.1093/hr/uhac016

**Published:** 2022-02-19

**Authors:** Xin Liu, Hao-Feng Liu, Hong-Liang Li, Xiu-Hong An, Lai-Qing Song, Chun-Xiang You, Ling-Ling Zhao, Yi Tian, Xiao-Fei Wang

**Affiliations:** 1 State Key Laboratory of Crop Biology, Shandong Collaborative Innovation Center of Fruit & Vegetable Quality and Efficient Production, Shandong Green Fertilizer Technology Innovation Center, College of Horticulture Science and Engineering, Shandong Agricultural University, Taian 271018, Shandong, China; 2 National Engineering Research Center for Agriculture in Northern Mountainous Areas, Agricultural Technology Innovation Center in Mountainous Areas of Hebei Province, Hebei Agricultural University, Baoding, Hebei, China; 3 Yantai Academy of Agricultural Sciences, Yantai, Shandong, China; 4 Beijing Academy of Forestry and Pomology Sciences, Beijing, China

## Abstract

Nitrate is the major nitrogen source for higher plants. In addition to serving as a nutrient, it is also a signaling molecule that regulates plant growth and development. Although membrane-bound nitrate transporter/peptide transporters (NRT/PTRs) have been extensively studied and shown to regulate nitrate uptake and movement, little is known about how these factors are regulated by the external nitrogen environment. Red-fleshed apple, whose coloration is determined by the transcription factor MdMYB10, had higher nitrate uptake efficiency than non-red-fleshed apple. Nitrate assimilation and utilization were higher in red-fleshed apple cultivars, and comparative transcriptome analysis showed that the expression of genes encoding the *NRT2s* was increased in red-fleshed apple. *In vitro* and *in vivo* experiments showed that MdMYB10 directly bound to the *MdNRT2.4-1* promoter to transcriptionally activate its expression, resulting in enhanced nitrate uptake. MdMYB10 also controlled nitrate reallocation from old leaves to new leaves through *MdNRT2.4-1*. Overall, our findings provide novel insights into the mechanism by which MdMYB10 controls nitrate uptake and reallocation in apple, which facilitates adaptation to a low-nitrogen environment.

## Introduction

Nitrogen is a main limiting factor for plant growth and development. In most plants, nitrate (NO_3_^−^) is the primary nitrogen source [1]. To be assimilated, nitrogen must be absorbed from the soil, converted into ammonium by nitrate reductase (NR) and nitrite reductase, and then converted into amino acids by glutamine synthetase and glutamine oxoglutarate aminotransferase [2]. Plants have evolved sophisticated mechanisms for nitrogen acquisition to promote their growth and development in various nitrogen environments.

Recent research on nitrate transporters has provided novel insights into the regulatory mechanisms of nitrate uptake and allocation. To adapt to the wide range of nitrate concentrations on land, plants have evolved two nitrate uptake pathways: the high-affinity transport system (HATS) and the low-affinity transport system (LATS) [3]. Chlorate resistant 1 (CHL1), also known as NRT1.1, was the first NRT1 transporter to be identified and belongs to the NITRATE TRANSPORT 1/PEPTIDE TRANSPORT (NRT1/PTR) family [4]. In *Arabidopsis*, there are 53 NRT1/PTR transporters, most of which are characterized as low-affinity nitrate transporters. In contrast to NRT1/PTR transporters, all NRT2 transporters are high-affinity nitrate transporters [5]. In *Arabidopsis*, there are seven NRT2 transporters, which participate in high-affinity nitrate uptake [6]. NRT2.1/NRT2.2 are involved in nitrate influx in nitrogen-starved plants [7], NRT2.4 plays an important role under nitrate-deprived conditions [8], NRT2.5 plays a key role in nitrate uptake into the phloem during nitrogen remobilization in adult plants [9], and NRT2.7 is responsible for nitrate loading during seed storage [10], indicating that NRT2s have different specialized roles in the regulation of nitrate uptake under low nitrate conditions.

In addition to being an essential macroelement, nitrate also serves as a signaling molecule that affects plant growth and development, and several genes have been identified that regulate these nitrate-dependent processes [11]. In addition to its activity as an NRT1 transporter, NRT1.1 also functions as a nitrate sensor, and CBL-INDEPENDENT PROTEIN KINASE 8 (CIPK8) and CIPK23 are involved in the phosphorylation and dephosphorylation of NRT1.1 in response to the external nitrate environment [12, 13].

Nitrate is absorbed from the soil and distributed through the plant; several processes are known to improve nitrogen use efficiency (NUE), including nitrogen uptake, translocation, assimilation, and remobilization [2]. Forward and reverse genetic studies have identified several downstream regulators that modulate nitrate uptake and utilization [10, 14, 15]. In *Arabidopsis*, *LOB DOMAIN-CONTAINING PROTEIN 37/38/39* (*LBD37/38/39*) are induced by nitrate and then inhibit nitrate uptake by repressing the expression of *NRTs* [16]. The NIN LIKE PROTEIN 7 (NLP7) protein exhibits nitrate-dependent subcellular localization and improves nitrate uptake by regulating *NRT1.1* expression [17, 18]. TEOSINTE BRANCHED 1, CYCLOIDEA, PCF (TCP)-DOMAIN FAMILY PROTEIN 20 (TCP20) interacts with NLP7 and directly binds to the *NRT2.1* promoter to regulate nitrogen assimilation and root development [19]. NITRATE REGULATORY GENE 2 (NRG2) interacts with NLP7 to regulate nitrate signals [20]. *NRT2.1* and *NRT2.2* are targets of TGACG SEQUENCE-SPECIFIC BINDING PROTEIN 1/4 (TGA1/4), the transcripts of which are upregulated by nitrate [21]. Using a systems biology approach, SQUAMOSA PROMOTER BINDING PROTEIN-LIKE 9 (SPL9) was identified as a regulator of the nitrate response that acts by altering the transcript levels of *NRT2.1* and other sentinel genes [22]. ELONGATED HYPOCOTYL5 (HY5), a bZIP transcription factor (TF), activates *NRT2.1* expression to regulate nitrate uptake [23]. MYB DOMAIN PROTEIN 59 (MYB59) binds directly to the *NRT1.5* promoter to regulate K^+^/NO_3_^−^ translocation in response to K^+^ deficiency [24]. *NITRATE-INDUCIBLE, GARP-TYPE TRANSCRIPTIONAL REPRESSOR 1/HYPERSENSITIVE TO LOW PI-ELICITED PRIMARY ROOT SHORTENING 1* (*NIGT1*/*HRS1*) is induced by nitrate and acts as a transcriptional repressor to negatively regulate *NRT2.1* expression [25]. In wheat, TaWabi5 is involved in nitrate acquisition by regulating the transcripts of TaNRT2s/NARs [26]. In rice, OsNAC42 promotes NUE by activating the expression of *OsNPF6.1* [27]. NITROGEN-MEDIATED TILLER GROWTH RESPONSE 5 (NGR5) interacts with a component of polycomb repressive complex 2 (PRC2) to regulate nitrogen-promoted tillering and enhance NUE [28]. The GROWTH-REGULATING FACTOR 4 (OsGRF4) TF promotes NUE by regulating NH_4_^+^ uptake and assimilation [29]. Members of the *AUXIN RESPONSE FACTOR* (*OsARF*) family are induced by auxin and improve NUE through the transcriptional activation of nitrate transporter and N-metabolism genes [30].

Improving NUE is an effective strategy for improving crop cultivation. This requires an understanding of the mechanism by which nitrate is assimilated and absorbed, and this information can be used to breed nitrogen-sensitive varieties. This is a common approach in agricultural production, and many quantitative trait loci associated with nitrate uptake, assimilation, and utilization have been identified. In rice, a single amino acid change in OsNRT1.1 leads to higher NUE in *indica* compared with *japonica* [31]. *OsNRT2.3* has two spliced isoforms, one of which increases nitrogen uptake through a regulatory motif that enhances the pH-buffering capacity of plants [32]. The natural allele of *OsNPF6.1* enhances nitrate uptake under low nitrogen supply [27]. Recent omics studies have revealed natural variation in *OsMYB61* [33]. *TCP DOMAIN PROTEIN 19* (*OsTCP19*) [34], *Dull Nitrogen Response 1* (*OsDNR1*) [30], and *OsGRF4* [29] have also been identified as associated with NUE. To date, most research on nitrogen uptake and utilization has been restricted to herbaceous plants, and little is known about the uptake and utilization of nitrogen in apple or other model perennial woody plants.

Red-fleshed apples produce red leaves and red-fleshed fruits in a process that is genetically determined by the *MdMYB10* locus. *MdMYB10* contains a 6-repeat enhancer in the promoter region and is a dominant allelic mutant corresponding to *MdMYB1* and *MdMYBA* [35, 36]. Compared with *MdMYB1*, the *MdMYB10* locus is dominant and produces higher transcript levels of *MdMYB10* [36]. In this study, red-fleshed apple cultivars were found to exhibit higher nitrate uptake efficiency. In addition, MdMYB10 was found to regulate nitrate uptake and nitrate reallocation from old leaves to new leaves. The molecular mechanism by which MdMYB10 regulates nitrate uptake and reallocation to adapt to different nitrogen environments was elucidated and discussed.

## Materials and methods

### Plant materials and growth conditions

To identify the role of the *MdMYB10* locus in the regulation of nitrogen uptake, 84 red-fleshed cultivars and 35 non-red-fleshed cultivars growing in the same orchard in Tai-An were examined. The leaves on new branches from the current year were collected from hybrid populations for further study in mid-July. Three hybrid populations of red-fleshed apple were used in this study. In one of the hybrid populations, “B9” (“Budagovsky, Bud-9”) and “YL” (“Jinshayilamu”), both of which contain a heterozygous *MdMYB10* locus (MYB10/myb10 genotype), were crossed. In the second hybrid population, the red-fleshed apple “Radiant” (MYB10/myb10) was crossed with the non-red-fleshed apple “Orin” (myb10/myb10). In the third cross, the red-fleshed apple “B9” (MYB10/myb10) was crossed with the non-red-fleshed apple “Royal Gala” (myb10/myb10). Ten red-fleshed trees and 10 non-red-fleshed trees with healthy and consistent growth were selected from each population, and the roots and leaves of these seedlings were collected at regular intervals and mixed for subsequent analyses.

To test the effect of the *MdMYB10* locus on the reallocation of nitrate from old to young leaves, 10 red-fleshed and non-red-fleshed trees of the “B9” × “Royal Gala” hybrid population were used, and three branches (40 cm long) from each hybrid population were collected for *in vitro* treatment. These branches were cultured in 0.5 mM KNO_3_ (low nitrate) and 5 mM KNO_3_ (high nitrate) nutrient solutions (MS medium prepared without nitrogen, with KNO_3_ added to the indicated concentrations and a K^+^ concentration up to 5 mM) for 10 days. The branches were grown at 23–25°C with a 16-h light/8-h dark cycle and a light intensity of 200 μmol m^−2^·s^−1^. The leaves of these branches were then collected and mixed for subsequent analyses.

To identify the role of the *MdMYB10* locus in regulating the transcripts of genes involved in nitrogen uptake, the seeds of a natural hybrid population of the red-fleshed apple “Radiant” were collected and stratified at 4°C for 30 days. After germination, the seedlings were cultivated in a nutrient solution for 30 days. Fifteen red-fleshed and 15 non-red-fleshed seedlings were exposed to a nitrogen deficiency treatment for 5 days, and then these seedlings were treated with 0.5 mM KNO_3_ and 5 mM KNO_3_ nutrient solutions (as described above) for 15 days. These seedlings were grown at 23–25°C with a 16-h light/8-h dark cycle and a light intensity of 200 μmol m^−2^·s^−1^. The roots and leaves of these seedlings were collected and mixed for subsequent analyses.

“Orin” apple calli were cultured on MS medium with 1.5 mg L^−1^ 2,4-D and 0.5 mg L^−1^ 6-BA in a continuously dark environment at 25°C. For nitrate treatment, calli were cultured in 0.5 mM KNO_3_ or 5 mM KNO_3_ medium (as described above) in the dark for 1 week and then treated with continuous light for 3 days at a light intensity of 200 μmol m^−2^·s^−1^.

*Arabidopsis* seeds were sown on MS medium. Four days after sowing, seedlings were transferred to culture in 0.5 mM KNO_3_ or 5 mM KNO_3_ medium for 10 days (as described above), and then their roots and shoots were collected for further study. *Arabidopsis* plants were grown at 19–22°C with a 16-h light/8-h dark cycle and a light intensity of 200 μmol m^−2^·s^−1^.

The basic nutrient solution contained 1.0 mM CaCl_2_, 1.0 mM KH_2_PO_4_, 1.0 mM MgSO_4_, 0.1 mM FeNa_2_EDTA, 50 μM MnSO_4_·H_2_O, 50 μM H_3_BO_3_, 0.05 μM CuSO_4_·5H_2_O, 0.5 μM Na_2_MoO_4_·2H_2_O, 15 μM ZnSO_4_·7H_2_O, 2.5 μM KI, and 0.05 μM CoCl·6H_2_O. Solutions with different nitrate concentrations were prepared by adding different volumes of 1.0 M KNO_3_ and 1.0 M KCl (to ensure that the final concentration of K^+^ remained the same) to the basic nutrient solution.

### Plasmid construction and genetic transformation

The coding sequences of *MdMYB10* were ligated into the pRI101-GFP vector. The MdMYB10-anti vector was also constructed, with the special fragments of *MdMYB10* ligated into the pRI101-GFP vector. The full-length cDNA sequences of *MdNRT2.4-1* were ligated into the pCXSN vector. To construct the MdNRT2.4-1-TRV vector, cDNA fragments of *MdNRT2.4-1* were ligated into the TRV vector. To clone the *MdNRT2.4-1* promoter, a 2-kb promoter sequence upstream of the *MdNRT2.4-1* start codon was ligated into the pXGUS-P vector. To construct the MdNRT2.4-1_pro_(MBS-1)-pAbAi and MdNRT2.4-1_pro_(MBS-1m)-pAbAi vectors, cDNA fragments of the MdNRT2.41 promoter were inserted into the pAbAi vector. The prokaryotic expression vector was constructed using pET32a, and the Y1H vector was constructed using pGAD. The primers used for gene cloning and vector construction are listed in Supplemental Table S1.

Transformation of apple calli and apple plantlets was carried out using *Agrobacterium tumefaciens* strain LBA4404. The transgenic materials were selected on medium that contained kanamycin (30 mg/L for apple calli and 50 mg/L for apple plantlets) or hygromycin B (15 mg/L for apple calli and 30 mg/L for *Arabidopsis*).

### Measurements of nitrate content, total nitrogen content, NR activity (NRA), ^15^N concentration, and nitrogen derived from fertilizer (NDFF)

**Nitrate content analysis:** Nitrate was measured using the salicylic acid method [37]. In brief, weighed samples (approximately 1.0 g for roots, leaves, or shoots of the hybrid population, branches, and seedlings; 1.5 g for calli) were placed in a test tube. Next, 10 ml of ddH_2_O was added, and the mixture was boiled for 1 h; 0.1 ml of supernatant liquid was placed into a 1.5-ml tube and mixed with 0.4 ml of salicylic acid–sulfate acid. After incubation at 25°C for 20 min, 9.5 ml of 8% NaOH solution was added, and the OD_410_ value was determined after the solution had cooled. A 0.1-ml volume of ddH_2_O was used as the control. An equation was used to calculate the nitrate content: Y (μmol g^−1^ FW) = 1000C·V/W/M (Y: nitrate content, C: nitrate concentration calculated by substituting OD_410_ into the regression equation, V: total volume of the extracted sample, W: weight of the sample, M: amount of substance).

**Total nitrogen content:** Plant samples were dried at 80°C before dry weight measurements. The dried samples (approximately 0.2 g) were milled and then digested with concentrated H_2_SO_4_ to determine total nitrogen content using a semi-automated Kjeldahl method (Tecator Kjeltec Auto 1030 Analyzer; Tecator) [38].

**NRA detection:** NR activity was measured using an *in vivo* assay [39]. Weighed samples (approximately 1.0 g) were incubated in 9 mL of 0.1 M phosphate buffer (pH 7.5) containing 1% propanol and 0.1 M KNO_3_. The samples were vacuum-infiltrated three times, and the tubes were stored at 30°C for 30 min. Nitrite was determined by adding 1 mL of 30% trichloroacetic acid. Test tubes were stored for 2 min, and 2 ml of supernatant was transferred into a new tube; 4 ml of 1% sulfanilamide was prepared with 3 M HCl, and 0.3 mL of 0.2% N-1-naphthyl- ethylene-diamine and 4 ml of 0.2% naphthalene were added. The sample was mixed well, stored for 30 min, and analyzed by colorimetry at 540 nm. NRA was expressed as nmol nitrite produced per hour per gram of dry weight (μmol h^−1^ g^−1^ FW).

^**15**^ **N Labeling Assay**: ^15^NO_3_^−^ (atom% ^15^N: 10.16%) was applied to each tree selected from the two hybrid populations. The roots and leaves of these trees were collected at regular intervals and mixed for subsequent analyses. Influx of ^15^NO_3_^−^ was calculated as previously described [34]. In brief, the samples (calli, seedlings, and *Arabidopsis*) were treated with 0.1 mM CaSO_4_ for 1 min, then with complete nutrient solution containing ^15^NO_3_^−^ (atom% ^15^N: 98.00%) for 5 min, and finally with 0.1 mM CaSO_4_ for 1 min. The samples (calli, leaves, roots, and shoots) were then collected separately. All samples were dried, and ^15^N content was determined using an isotope-ratio mass spectrometry system (Thermo Scientific, USA).

**NDFF:** The percentages of NDFF in the apple plants were calculated using the following formula: NDFF = (As−A0)÷(AF−A0) × 100% (As: ^15^N in sample, AF: ^15^N in fertilizer [atom% ^15^N: 10.16% or 98.00%], A0: natural abundance of ^15^N [0.3663%]).

### RNA extraction, RT-PCR, and qRT-PCR assays

Total RNA was extracted from “Orin” calli, non-red-fleshed apple plants, and red-fleshed apple plants using the RNAplant Plus Reagent (Tiangen, Beijing, China) according to the manufacturer’s instructions. First-strand cDNA was synthesized using the corresponding kit (TaKaRa, Dalian, China). qPCR was performed with SYBR-Green PCR Master Mix using the iCycler iQ5 system (Bio-Rad, Hercules, CA, USA). Relative gene expression was calculated using *18S* as the internal control gene. Three biological replicates were performed for each experiment. The primers used for qRT-PCR are listed in Table S1.

### Electrophoretic mobility shift assay (EMSA)

The EMSA was performed as previously described by Xie et al. [40]. *MdMYB10* was inserted into pET32a. The MdMYB1-HIS recombinant protein was expressed and then purified. The probe of the *MdNRT2.4* promoter was labeled according to the manufacturer’s instructions. MdMYB10-HIS was then incubated with the relative reaction components as described previously [40]. After the binding reaction, the complexes were separated, electrotransferred, and detected according to the manufacturer’s instructions. The binding specificity of AtPAP1/2-HIS with the AtNRT2.4 promoter was examined using a similar method, and the primers used for EMSA are listed in Table S1.

### Chromatin immunoprecipitation (ChIP) qPCR analysis

35S::MdMYB10-GFP and 35S::GFP transgenic apple calli and 35S::AtPAP1-GFP, 35S::AtPAP2-GFP, and 35S::GFP transgenic *Arabidopsis* were used for the ChIP-qPCR analysis. The ChIP analysis was performed as described previously [40]. The primers used for Chip-PCR are listed in Table S1.

### Yeast one-hybrid (Y1H) assay

Y1H assays were performed using the Yeastmaker Yeast Transformation System 2 (Clontech, Palo Alto, CA, USA) according to the manufacturer’s instructions. The cDNA of the MdMYB10 sequence was amplified and inserted into pGAD to construct pGAD-MdMYB10 vector, and MdNRT2.4-1 promoter was inserted in to the pABAi to construct pABAi-MdNRT2.4-1pro vector (see Table S1). They were then co-transformed into yeast Y1H Gold (Clontech, Palo Alto, CA, USA). The yeast was grown on SD base/−Leu/−Ure selection medium and then transferred to selection medium supplemented with aureobasidin A (AbA) to determine the interactions between MdMYB10 and the *MdNRT2.4-1* promoter.

### Transient expression assays

Transient expression assays in apple calli were used to verify the functions of MdMYB10 and MdNRT2.4-1 in the regulation of nitrate uptake. The resultant recombinant plasmid MdNRT2.4-1 + TRV2 was genetically introduced into MdMYB10-GFP and MdMYB10-anti transgenic “Orin” apple calli by *Agrobacterium*-mediated transformation.

Transient expression assays in *N. benthamiana* leaves were performed to verify the transcriptional activation of *MdNRT2.4-1* by MdMYB10*.* The *MdNRT2.4-1* promoter (2 kb upstream of the start codon) was inserted into the pGreenII 0800-LUC vector to construct MdNRT2.4-1pro:Luc, and the full length of *MdMYB10* was inserted into the pGreenII 62-SK vector to construct 35Spro:MdMYB10. The recombinant plasmids (35Spro:MdMYB10 + MdNRT2.4-1pro:Luc, 35Spro + MdNRT2.4-1pro:Luc or 35Spro + pGreenII 0800-LUC) were transformed into *N. benthamiana* leaves by *Agrobacterium*-mediated transformation, and the LUC images were collected with a charge-coupled device imaging apparatus.

### Measurement of anthocyanin content

Anthocyanins were extracted with a methanol-HCl method and detected as described by Lee and Wicker [41].

### Promoter–GUS analysis

The recombinant plasmid MdNRT2.4-1_pro_::GUS was genetically introduced into MdMYB10-GFP and MdMYB10-anti transgenic “Orin” apple calli by *Agrobacterium*-mediated transformation. The transgenic calli were separately transferred to medium containing 5 or 0.5 mM KNO_3_ for 1 h. After treatment, histochemical staining was performed to detect GUS activity, as described by Xie et al. [40]. GUS antibody was used to detect the abundance of GUS protein. MdNRT2.4-1_pro_::GUS was also genetically introduced into *Arabidopsis*, and histochemical staining was performed using the same method [40].

### Data analysis

We used three biological and three technical replicates for all measurements, and results are reported as mean values in the text and as mean values ± SD in the figures and tables. Statistical significance was determined by Tukey’s test performed in DPS v9.50 software [42].

## Results

### The *MdMYB10* locus is involved in regulating nitrate uptake and usage

**Figure 1:**
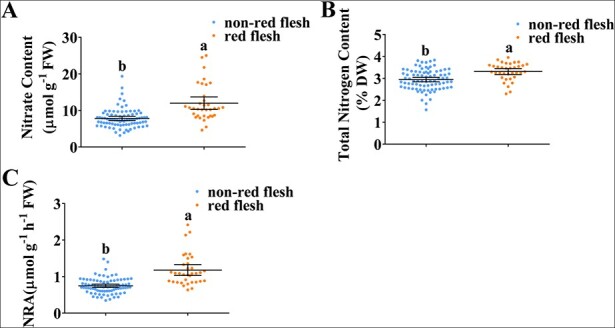
Effect of red-fleshed cultivars on the regulation of nitrate usage. The nitrate content (A), total nitrogen content (B), and NRA (C) were determined. Data are presented as mean ± SD, and significant differences between apple calli lines were determined using Tukey’s test. Different letters represent significant differences.

Apples can be divided into red-fleshed cultivars and non-red-fleshed cultivars. Here, 35 red-fleshed cultivars and 84 non-red-fleshed cultivars were screened for their nitrate content, total nitrogen content, and NR activity. Overall, nitrate content, total nitrogen content, and NRA were higher in red-fleshed cultivars than in non-red-fleshed cultivars (Fig. 1), suggesting that red-fleshed cultivars have higher nitrate uptake and usage than non-red-fleshed cultivars.

To determine whether there was a direct genetic link between the red-fleshed *MdMYB10* locus and nitrate usage, two hybrid populations were generated. Five red and five non-red-fleshed individuals were then randomly chosen from each hybrid population (Fig. S1A–B). The red-fleshed hybrids produced more *MdMYB10* transcripts and accumulated more anthocyanins than the non-red-fleshed hybrids (Fig. S1C–F). Subsequently, samples of roots, leaves, and fruits of red- and non-red-fleshed subpopulations were collected and mixed to measure nitrogen uptake. Red-fleshed sub-populations had higher nitrate content, total nitrogen, NDFF, and NRA than the non-red-fleshed sub-populations in the two hybrid populations (Table S2). A natural hybrid population of the red-fleshed apple Radiant was also examined; 15 red-fleshed and 15 non-red-fleshed samples were collected. The nitrogen content was measured, and similar results were obtained (Fig. S2). Overall, these results indicate that the red flesh locus *MdMYB10* mediates nitrate use efficiency**.**

**Figure 2:**
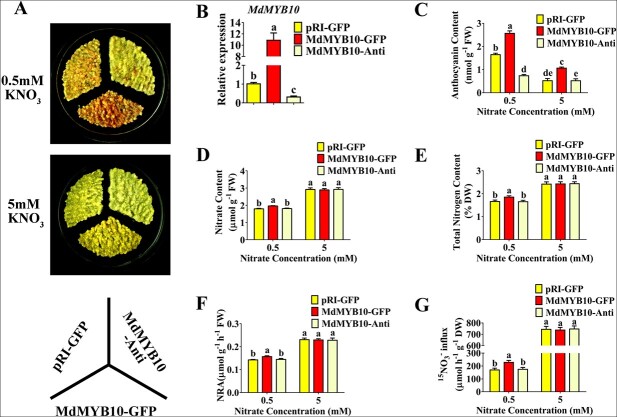
MdMYB10 controls nitrate uptake. (A) Anthocyanin accumulation phenotype in wild-type and MdMYB10 transgenic apple calli under low (0.5 mM) and high (5 mM) nitrate treatment. (B) The relative expression of *MdMYB10* in transgenic apple calli. (C) Anthocyanin content in wild-type and MdMYB10 transgenic apple calli under low (0.5 mM) and high (5 mM) nitrate treatment. (D)–(G) Determination of nitrate content (D), total nitrogen content (E), NRA (F), and ^15^N accumulation (G) in wild-type and transgenic apple calli. In (B)–(G), data are presented as mean ± SD, and significant differences between red-fleshed and non-red-fleshed fruits were determined using Tukey’s test. Different letters represent significant differences.

The expression of *MdMYB10* was induced by low nitrate conditions (Fig. S3). To determine whether the MdMYB10 TF directly regulates nitrate use efficiency, the overexpression vector 35S::MdMYB10-GFP and the suppression vector 35S::MdMYB10-anti were constructed for genetic transformation. The empty 35S::GFP (pRI-GFP) vector was used as the control. Three types of transgenic “Orin” calli, 35S::MdMYB10-GFP, 35S::MdMYB10-anti, and 35S::GFP, were obtained (Fig. 2A–B). These samples were then treated with low and high nitrate. The accumulation of anthocyanins was higher in 35S::MdMYB10-GFP and lower in 35S::MdMYB10-anti transgenic calli compared with the 35S::GFP control, especially under low nitrate conditions (Fig. 2A, C), indicating that *MdMYB10* was successfully expressed in transgenic apple calli. The nitrate content, total nitrogen content, and NRA were much higher in 35S::MYB10-GFP transgenic calli than in the 35S::GFP control under low nitrate conditions (Fig. 2D–F). After treatment with ^15^N-labeled nitrate, ^15^N accumulation was higher in 35S::MdMYB10-GFP transgenic calli compared with the 35S::GFP control under low nitrate conditions (Fig. 2G). There was no significant difference in nitrate use efficiency between control and transgenic apple calli under high nitrate conditions (Fig. 2D–G), indicating that MdMYB10 regulates nitrate uptake under low nitrate conditions.

### The *MdMYB10* locus in red-fleshed apple is involved in the regulation of genes associated with nitrate usage

**Figure 3:**
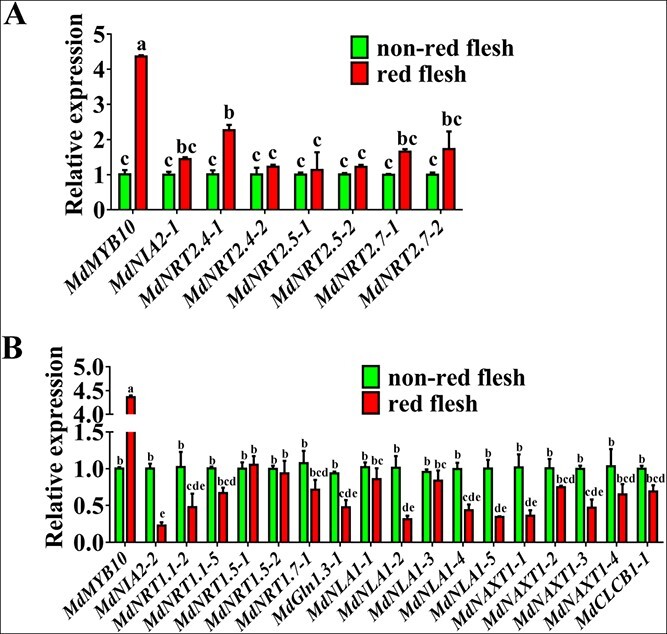
The *MdMYB10* locus is involved in regulating the abundance of transcripts involved in nitrate uptake. (A)–(B) Quantitative real-time PCR (qRT-PCR) analysis was used to detect the transcript abundance of genes involved in nitrate transport, uptake, and assimilation. Data are presented as mean ± SD of more than 10 red-fleshed or non-red-fleshed apples, and significant differences between apple calli lines were determined using Tukey’s test. Different letters represent significant differences.

To elucidate the molecular mechanism by which the *MdMYB10* locus in red-fleshed apple regulates nitrate usage, previously collected RNA-seq data on 39,875 genes were analyzed [43]. GO functional classification analysis of genes related to nitrate uptake and assimilation showed that only the expression of *NRT2* and *NIA* was upregulated in red-fleshed apple, and the expression of most *NRT1* family genes was downregulated (Table S3). The expression levels were then verified by quantitative real-time PCR (qRT-PCR) analysis, which showed that expression of *MdNRT2.4-1* and *MdNIA2-1* was upregulated (Fig. 3A), whereas transcript levels of genes related to nitrate transport (*MdNRT1s*), nitrate assimilation (*MdNIA2-2*), nitrogen metabolism (*MdGln1.3-1*), nitrate storage (*MdNAXT1s*, *MdCLCB1-1*), and nitrate signaling (*MdNLA1s*) were downregulated in red-fleshed apple (Fig. 3B).

The hybrid population experiment revealed changes in the expression of nitrate-responsive genes. *MdMYB10* transgenic materials were used to determine whether *MdMYB10* was responsible for the observed changes in the transcription of these genes. The 35S::MdMYB10-GFP transgenic calli exhibited much higher expression of *MdNRT2s* and *MdNIA2-1* compared with the control in the low nitrate treatment (Fig. S4A). The transcript levels of genes related to nitrate transport (*MdNRT1s*), nitrate assimilation (*MdNIA2-2*), nitrogen metabolism (*MdGln1.3-1*), nitrate storage (*MdNAXT1s*, *MdCLCB1-1*) and nitrate signaling (*MdNLA1s*) were not upregulated in the 35S::MdMYB10-GFP transgenic calli (Fig. S4B). These results indicated that MdMYB10 induced the expression of *MdNRT2s* and *MdNIA2-1* in a nitrate-dependent manner.

### MdMYB10 directly binds to the *MdNRT2.4-1* promoter and activates its transcription

**Figure 4:**
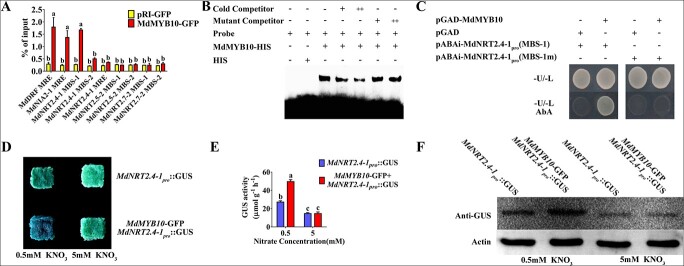
MdMYB10 specifically binds to the promoter of *MdNRT2.4-1*. (A) *In vivo* ChIP-PCR assays for detecting the enrichment of nitrate-associated genes. The MdMYB10-DNA complex was co-immunoprecipitated from *MdMYB1-GFP* transgenic apple calli using anti-GFP antibody. pBIN-GFP was used as a negative control. (B) ESMA assay for detecting the interaction between MdMYB10-HIS and the *cis*-elements of the *MdNRT2.4-1* promoter. pET32a-HIS was used as a negative control. (C) Y1H assay to detect the interaction between MdMYB10-HIS and the *MdNRT2.4-1* promoter; the pGAD vector was used as a negative control. (D–F) GUS staining was used to detect GUS activity in the transgenic apple calli. The means and standard deviations were calculated from the results of three independent experiments.

The expression of *MdNRT2s* and *MdNIA2-1* was induced by MdMYB10 in the red-fleshed apple and MdMYB10 transgenic calli. To examine whether MdMYB10 directly regulates the expression of these genes, their promoter regions located 2000 bp upstream of the initiation codon were searched for *cis*-elements, such as the MYB recognition element (MRE) and the MYB binding site (MBS). One or several MRE or MBS sites were identified in the promoter regions (Fig. S5A). Next, *in vivo* ChIP-PCR assays were performed using transgenic apple calli overexpressing 35S::MdMYB10-GFP with anti-GFP antibody. The MdMYB10-GFP fusion protein was noticeably enriched in the *cis*-element regions of the *MdNIA2–1* and *MdNRT2.41* promoters (Fig. 4A).

EMSA was performed to confirm the binding of MdMYB10 to these *cis*-elements. MdMYB1-HIS fusion proteins were expressed in bacteria and then purified. A specific DNA-MdMYB10 protein complex was detected when a *cis*-element-containing oligonucleotide was used as the labeled probe for *MdNIA2-1* (MRE) and *MdNRT2.4-1* (MBS-1) but not for probes targeting the other *MdNRT2s* (Fig. S5B). Next, competition-binding experiments were carried out using the probes for *MdNIA2-1* and *MdNRT2.4-1*. There was a reduction in the intensity of the shift when increasing amounts of unlabeled MBS-1 competitor probe were added. However, this competition was not observed when an unlabeled MBS-1 mutant probe was used (Fig. 4B), indicating a direct interaction between MdMYB10 and *MdNRT2.4-1*. For *MdNIA2-1* promoters, competition was not observed when an unlabeled MRE competitor probe was added (Fig. S5C), indicating a non-specific binding of MdMYB10 to *MdNIA2-1* promoters. ChIP-PCR, coupled with the EMSA results, suggested that an MdMYB10-interacting partner, rather than MdMYB10 itself, directly interacted with the *MdNIA2-1* promoter.

Y1H assays were performed to verify the interaction between MdMYB10 and the *MdNRT2.4-1* promoter. Yeast containing pGAD-MdMYB1 and pAbAi-MdNRT2.4-1pro (MBS-1) exhibited normal growth on −U/−L screening medium with 50 mg/L AbA, but yeast containing pGAD and pAbAi-MdNRT2.4-1pro (MBS-1) did not show normal growth under these conditions (Fig. 4C, left panel). A promoter containing a mutant MBS *cis*-element was also engineered into the pAbAi vector, and yeast containing pGAD-MdMYB1 and pAbAi-MdNRT2.4-1pro (MBS-1m) was not capable of normal growth on −U/−L screening medium with 200 mg/L AbA (Fig. 4C, right panel). These results confirmed that MdMYB10 interacted directly with the *MdMNRT2.4-1* promoter and that the MBS element was essential for this interaction.

MdMYB10 interacted with the *MdNRT2.4-1* promoter and transcriptionally activated its expression (Fig. S6). To further examine whether MdMYB10 transcriptionally activated the *MdNRT2.4-1* promoter, the recombinant pNRT2.4-1::GUS plasmid was constructed and transformed into apple calli, and 35S::MdMYB10-GFP was then co-transformed into the pNRT2.4-1::GUS calli. The co-transformed calli containing pMdNRT2.4-1::GUS and 35S::MdMYB10-GFP exhibited much higher GUS activity and GUS protein accumulation than calli containing pMdNRT2.4-1::GUS alone under low nitrate conditions. However, no difference in GUS activity or GUS protein accumulation was observed under high nitrate conditions (Fig. 4D–F), suggesting that the regulation of *MdNRT2.4-1* transcriptional activity was mediated by MdMYB10 through a nitrate-dependent pathway.

### MdMYB10 partially modulates nitrate uptake and reallocation through *MdNRT2.4-1*

The above results show that MdMYB10 induced the expression of *MdNRT2.4-1* and increased nitrate uptake. To test whether MdMYB10 regulated nitrate usage through *MdNRT2.4-1*, the role of *MdNRT2.4-1* in regulating nitrate uptake was characterized. Phylogenetic analysis showed that MdNRT2.4-1 was homologous to *Arabidopsis* NRT2.4, a transmembrane domain protein (Fig. S7A). The MdNRT2.4-1 protein has two AE motifs, similar to the motif of the OsNRT2.3b protein, which may function as a cytosolic pH sensor and regulatory domain [44] (Fig. S7B). To examine the temporal and spatial expression of *MdNRT2.4-1*, the reporter vector pMdNRT2.4-1::GUS was constructed and genetically transformed into *Arabidopsis*, and histochemical staining was performed. *MdNRT2.4-1* was mainly expressed in the epidermis of the lateral roots and hypocotyl, as well as in the primary veins of the leaves and shoots (Fig. S8A). Under high nitrate conditions, GUS activity was repressed (Fig. S8B), demonstrating that the expression of *MdNRT2.4-1* was negatively regulated under high nitrate conditions.

Subsequently, the overexpression vector 35S::MdNRT2.4-1 was constructed and genetically transformed into apple calli and *Arabidopsis* (Fig. S9A–B). Transgenic calli and *Arabidopsis* were then screened for nitrate content, NRA, total nitrogen, and ^15^N labeling. 35S::MdNRT2.4-1 overexpression calli and *Arabidopsis* had higher nitrate usage than the control under low, but not high, nitrate concentrations (Fig. S9C–J). These results indicate that MdNRT2.4-1 played a key role in nitrate usage in response to low nitrate concentrations but not high nitrate concentrations.

**Figure 5:**
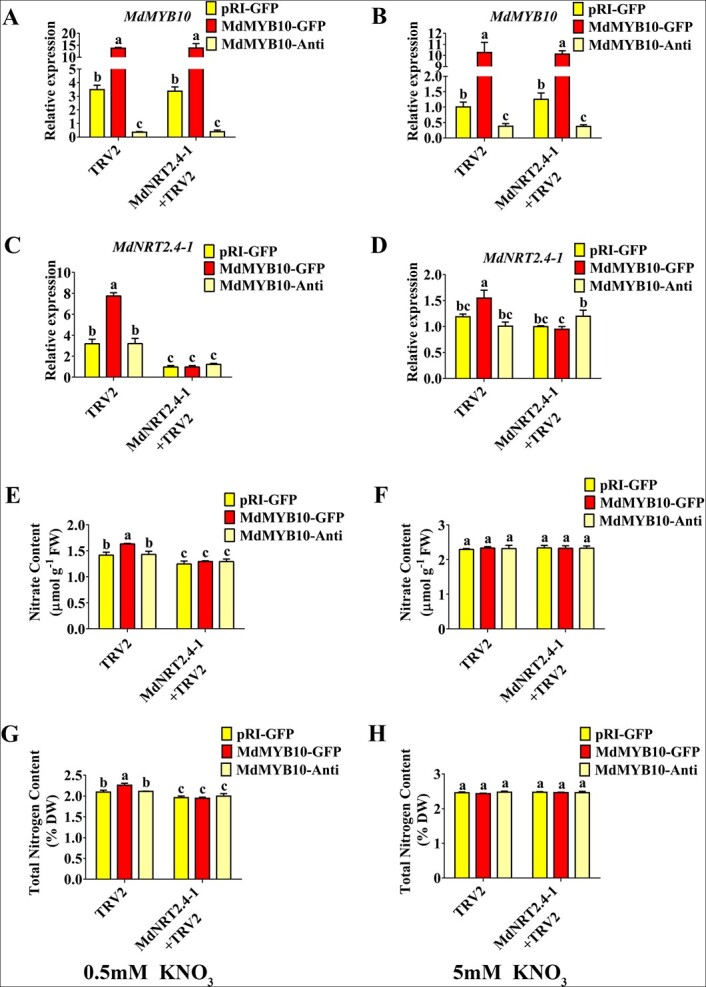
MdNRT2.4-1 participates in MdMYB10-modulated nitrate uptake. (A–D) Transcript levels of *MdMYB10* (A, B) and *MdNRT2.4-1* (C, D) in MdMYB10 transgenic calli under different nitrate treatments. (E–H) Detection of the nitrate and total nitrogen content in *MdMYB10* transgenic calli under different nitrate treatments. In (A)–(H), data are presented as mean ± SD, and significant differences between red-fleshed and non-red-fleshed fruits were determined using Tukey’s test. Different letters represent significant differences.

To better understand the relationship between *MdMYB10* and *MdNRT2.4-1*, a tobacco rattle virus (TRV)-based TRV-MdNRT2.4-1 vector was used to verify the functions of MdMYB10 and MdNRT2.4-1 in the regulation of nitrate uptake. TRV-MdNRT2.4-1 and TRV control were separately co-transformed into 35S::MdMYB10, 35S::MdMYB10-anti, and wild-type calli. Next, qPCR assays were performed to measure expression. There was no difference in the expression of *MdMYB10*; however, the expression of *MdNRT2.4-1* was repressed in TRV-MdNRT2.4-1 co-transformed calli compared with the TRV control (Fig. 5A–D). The nitrate level and total nitrogen content were measured in the co-transformed calli, and the results showed that *MdMYB10* overexpression enhanced nitrate and total nitrogen content under low, but not high, nitrate conditions. However, this effect was repressed in MdMYB10/TRV-MdNRT2.4-1 co-transformed calli (Fig. 5E–H). These results demonstrated that the control of nitrate uptake by MdMYB10 was partially, if not completely, dependent on *MdNRT2.4-1* in apple calli.

### Red-fleshed apple regulates nitrate reallocation from old to young leaves

**Figure 6:**
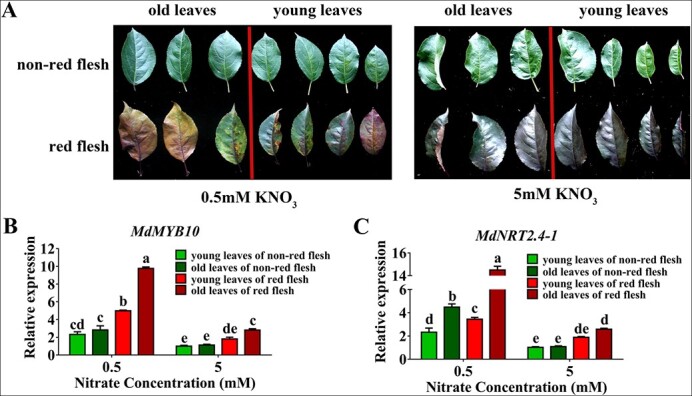
Expression patterns of *MdMYB10* and *MdNRT2.4-1* from old to young leaves. (A) Photos of old and young leaves from red-fleshed and non-red-fleshed hybrid trees that were treated with different concentrations of nitrate. (B)–(C) Transcript levels of *MdMYB10* (B) and *MdNRT2.4-1* (C) in old and young leaves from red-fleshed and non-red-fleshed hybrid trees under different nitrate treatments. In (B) and (C), data are presented as the mean ± SD of more than five trees with red-fleshed or non-red-fleshed fruits. Significant differences between red-fleshed and non-red-fleshed fruits were determined using Tukey’s test. Different letters represent significant differences.

*Arabidopsis NRT2.4* is expressed in or close to the phloem of leaves and participates in nitrate export from leaves only under nitrogen starvation conditions [8]. Red apple shows premature aging under nitrate-deficient conditions (Fig. 6A). In addition, both *MdMYB10* and *MdNRT2.4-1* are low nitrate-inducible genes (Fig. S10), suggesting that MdMYB10 might play a role in regulating nitrate remobilization from source to sink tissues through *MdNRT2.4-1* under low nitrate conditions. To test this hypothesis, expression analysis was performed in the red-fleshed and non-red-fleshed hybrid populations. The expression of *MdMYB10*, *MdNIA2*, and other *MdNRT2s* was higher in the red-fleshed population than in the non-red-fleshed population under nitrate-deficient conditions, and *MdNRT2.4-1* and *MdMYB10* exhibited similar expression patterns (Fig. 6B–C; Fig. S11).

**Figure 7:**
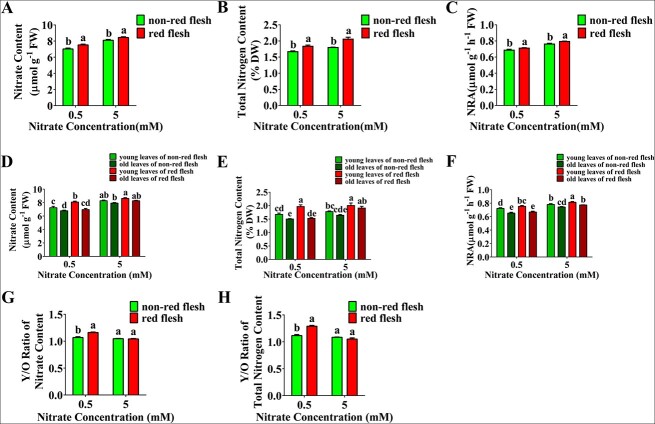
Regulation of nitrate reallocation from old to young leaves in red-fleshed apple. (A)–(C) Detection of nitrate content (A), total nitrogen content (B), and NRA (C) of leaves from red-fleshed and non-red-fleshed hybrid trees under different nitrate treatments. (D)–(F) Detection of nitrate content (D), total nitrogen content (E), and NRA (F) in old and young leaves from red-fleshed and non-red-fleshed hybrid trees under different nitrate concentrations. (G)–(H) The Young/Old ratio (Y/O ratio) of total nitrate (G) and nitrogen content (H) in whole young and old leaves of the red and non-red-fleshed populations. In (A)–(H), data are presented as the mean ± SD of more than nine replicates. Significant differences between calli lines were determined using Tukey’s test. Different letters represent significant differences.

The shoots of the two hybrid populations were treated with nitrate *in vitro* to test nitrate remobilization. Nitrate usage was higher in the red-fleshed population than in the non-red-fleshed population under both low and high nitrate conditions (Fig. 7A–C). The leaves were then divided into old leaves (lower leaves) and young leaves (upper leaves), and the nitrate uptake and assimilation were separately tested in these leaf samples. Young leaves accumulated more nitrate and total nitrogen than old leaves in both the red-fleshed population and non-red-fleshed population (Fig. 7D–F). Then, the total nitrate and nitrogen content in the whole young and old leaves were tested, and the Young/Old ratio (Y/O ratio) was calculated, which showed greater total nitrate and nitrogen accumulation in the young leaves in the red-fleshed population than in the non-red-fleshed population under low nitrate conditions (Fig. 7G–H). These findings, coupled with the results of the previous expression analysis, suggest that nitrate reallocation from old leaves to young leaves is enhanced in red-fleshed apple, possibly via *MdNRT2.4-1*.

**Figure 8:**
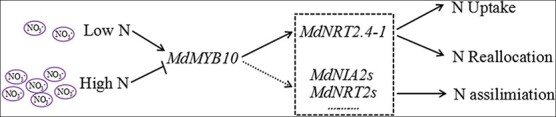
A model of how MdMYB10 transcriptionally activates *MdNRT2.4–1* and related genes to regulate nitrogen usage under various nitrogen environments.

We next asked whether the observed activity of MdMYB10 and its homologs in nitrate uptake regulation was conserved. AtPAP1/2, homologs of MdMYB10, were also functionally identified in *Arabidopsis*. PAP1 and PAP2 transgenic *Arabidopsis* [43] were used. First, the *cis*-elements in the promoter region of *AtNRT2.4* were analyzed, and three MBSs (I, II, and III) were identified (Fig. S12A). Next, *in vivo* ChIP-PCR assays were performed using transgenic *Arabidopsis* overexpressing either 35S::PAP1/2-GFP or 35S::GFP. There was clear enrichment of the PAP1-GFP fusion protein in the MBS-I *cis*-element, and the PAP2-GFP fusion protein was clearly enriched in the MBS-I/III *cis*-elements of the *AtNRT2.4* promoter (Fig. S12B). The physical binding of AtPAPs with *AtNRT2.4* was then demonstrated by an EMSA assay (Fig. S12C–E), indicating that the role of MdMYB10 in the regulation of nitrate uptake is conserved in different species.

## Discussion

Nitrate is an essential nutrient for crop production. The NRT gene family is involved in nitrate uptake from soil [5], which improves NUE and determines crop yields. In rice, polymorphism in NRT genes may affect the transport activity of NRT and ultimately increase overall NUE [31, 44]. There is thus a need to characterize how the genes that encode these transporters are transcriptionally regulated by TFs.

The correlation between transcripts of nitrate transporter genes and nitrate uptake activity indicates that transcriptional regulation plays a crucial part in the regulation of nitrate uptake. Our findings showed that high expression of *MdMYB10* was associated with high nitrate usage in red-fleshed fruits relative to non-red-fleshed fruits (Fig. 1). RNA-seq, biochemical experiments, and transgenic calli were used to demonstrate that red-fleshed apple exhibits improved nitrogen usage through the regulation of a variety of genes related to nitrate uptake (*MdNRT2s*) and assimilation (*MdNIA2s*), in addition to *MdNRT2.4-1* (Fig. 3; Fig. 4; Fig. S4; Table S2). The results of this research will aid the development of new apple cultivars with improved nitrate usage by increasing nitrate uptake and assimilation.

In higher plants, many TFs have been shown to modulate nutrient uptake and assimilation [10, 15]. However, few MYB TFs identified to date are known to modulate nitrate uptake and assimilation in higher plants, especially in fruit trees. MYB59 in *Arabidopsis* and MYB61 in rice have been identified as key regulators of nitrogen utilization [24, 33]. In *Arabidopsis*, expression of the MYB TF *PAP1* and its homologs is induced in response to nitrogen starvation, and these MYB TFs are involved in anthocyanin pigment metabolism and malate accumulation [36, 43]. However, whether these MYB TFs also participate in nitrate signaling remains unclear. We found that MdMYB10 enhanced nitrate usage by regulating nitrate uptake and assimilation in apple (Fig. 2; Fig. 3) and may function similarly in *Arabidopsis* (Fig. S12), indicating that MYB TFs play key roles in nitrate uptake and utilization. There were no statistically significant differences in nitrate uptake and assimilation in the MdMYB10-anti calli, suggesting that downregulation of *MdMYB10* is insufficient to significantly inhibit *MdNRT2.4-1* expression and nitrate uptake (Fig. 2D–G; Fig. 5C–D). This may reflect the functional redundancy of MdMYB10 with its homologs, which have a compensatory effect on the regulation of target gene expression. Transient expression assays showed that reducing transcripts of MdNRT2.4-1 inhibited MdMYB10 overexpression-enhanced nitrate and total nitrogen content (Fig. 5), demonstrating that control of nitrate uptake and utilization by MdMYB10 was partially, if not completely, dependent on MdNRT2.4-1 in apple.

Carbon and nitrogen metabolic pathways can have both antagonistic and synergistic effects on plants; thus, the homeostatic balance of carbon and nitrogen metabolism is important for promoting plant growth and development, as well as adaptation to fluctuating light and soil nitrogen environments [45, 46]. However, little is known about how shoot photosynthetic carbon assimilation and root inorganic nitrogen uptake are coordinated. Until recently, *Arabidopsis* light-induced HY5 protein was thought to activate the expression of *NRT2.1* to improve nitrate uptake [23]. *PAP1* (or *MdMYB10*) has been shown to be the target of AtHY5 (or MdHY5) in *Arabidopsis* and apple [47, 48]. Given that MdHY5 interacts with the *MdMYB10* promoter and MdMYB10 enhances nitrate uptake by regulating high-affinity nitrate transporters (Fig. 3), PAP1 (or MdMYB10) may play an important role in AtHY5-mediated (or MdHY5-mediated) nitrate uptake.

In addition to nitrate uptake, the redistribution of nitrate can also improve nitrate use efficiency. Several genes have been determined to play a role in nitrate reallocation [49]. NRT1.7, NRT1.11, and NRT1.12 are low-affinity transporters responsible for the source-to-sink remobilization of nitrate from mature leaves to new leaves with high N demand in response to different nitrate conditions [32, 50]. NRT2.4 is a high-affinity nitrate transporter expressed in phloem parenchyma cells that controls the absorption of nitrate into the phloem during nitrate remobilization under nitrogen starvation conditions [8]. Nitrogen deficiency accelerates the senescence of old leaves, which enhances nitrate remobilization from sources (old leaves) to sinks (new leaves) [51, 52]. In rice, NAC DOMAIN CONTAINING PROTEIN (OsNAP) interacts with ABA to regulate senescence and nutrient remobilization [53]. Our study also showed that red-fleshed apple exhibits a premature aging phenotype in old leaves (Fig. 6A). Compared with the wild type, red-fleshed mutants showed greater nitrate accumulation in the young leaves (Fig. 7). Overall, these data suggest that NRT2.4 in source leaves is involved in nitrate remobilization under nitrogen starvation conditions and that MdMYB10 mediates this process. Thus, plants use different nitrate allocation strategies to cope with different nitrate conditions.

The influx and assimilation of nitrate depend on cytosolic pH homeostasis. In rice, the nitrate transporter OsNRT2.3b contains regulatory AE motifs on the cytosolic side, enabling nitrate transport activity to be switched on or off through a pH-sensing mechanism [44, 54]. High *OsNRT2.3b* expression enhances pH-buffering capacity and increases nitrate uptake. In apple, MdNRT2.4-1 contains a similar pH-sensing AE domain [44] (Fig. S7). MdMYB10 has previously been shown to improve malate accumulation by regulating the vacuolar membrane H^+^-ATPase, which contributes to enhanced malic accumulation and elevated acidity in the vacuole [43]. This leads to an increase in cytoplasmic pH, which increases the transport activity of AE-motif-containing NRTs [44]. Expression analysis revealed that nitrate starvation induced *MdNRT2.4-1* expression and high nitrate reduced *MdNRT2.4-1* expression. Elevated vacuolar membrane H^+^-ATPase mediated by MdMYB10 can also supply energy for ion storage, increasing the nitrate content in the vacuole [43, 55]. Anthocyanins in edible plant organs are beneficial to human health, and their acidity contributes to taste and flavor. Many studies have shown that red coloration is regulated by MYB TFs [56]. Given that these MYB proteins modulate anthocyanin biosynthesis, malate accumulation, and osmotic stress [56], cultivars with excellent quality, fruit color, and acidity and higher nitrate use efficiency could be bred to improve the nutritional and market value of red-fleshed apples [57, 58]. Based on the findings in our study and the results of previous studies, a mechanism was proposed to explain how MdMYB10 and its homologs regulate nitrogen uptake reallocation as well as nitrate assimilation in response to various nitrogen environments (Fig. 8). The extent to which MdNRT2.4-1 regulates MdMYB10-enhanced nitrate uptake and usage remains to be clarified in future research.

## Supplementary Material

Web_Material_uhac016

## Data Availability

All relevant data can be found within the paper and its supporting materials.
