# Supplementary material for: MdMYB10 affects nitrogen uptake and reallocation by regulating the nitrate transporter MdNRT2.4-1 in red-fleshed apple
Source: Hortic Res. 2022 Feb 19;9:uhac016. doi: 10.1093/hr/uhac016 (PMC9016894; doi:10.1093/hr/uhac016)
Supplement: Web_Material_uhac016 [file web_material_uhac016.docx]

**
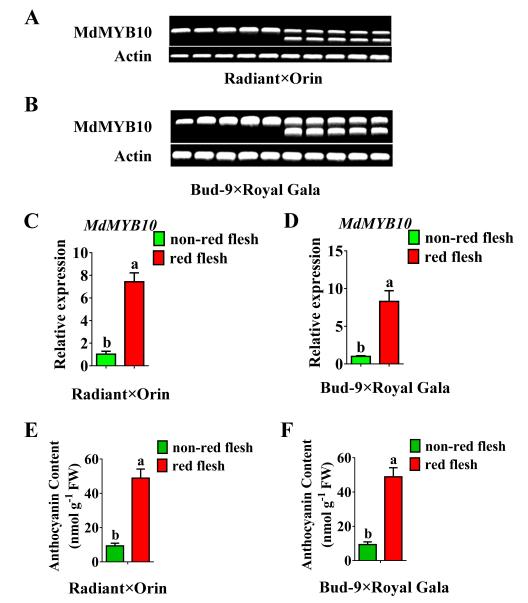
**

**Figure S1.** Identification of the hybrid populations from red flesh and non-red flesh apple cultivars.

(A)-(B) Identification of the genotype of the hybrid populations from two hybrid populations. Five red and five non-red flesh individuals were randomly chosen from each hybrid population. (C)-(D) Transcript levels of MdMYB10 in the red flesh hybrids compared with the non-red flesh apple hybrids. (E)-(F) Anthocyanin accumulation in the red flesh hybrids compared with the non-red flesh apple hybrids. For (C)-(F), data were mean±SD, and significant differences were determined using Tukey’s test, and different letters represent significant difference (a, b, c et al.).


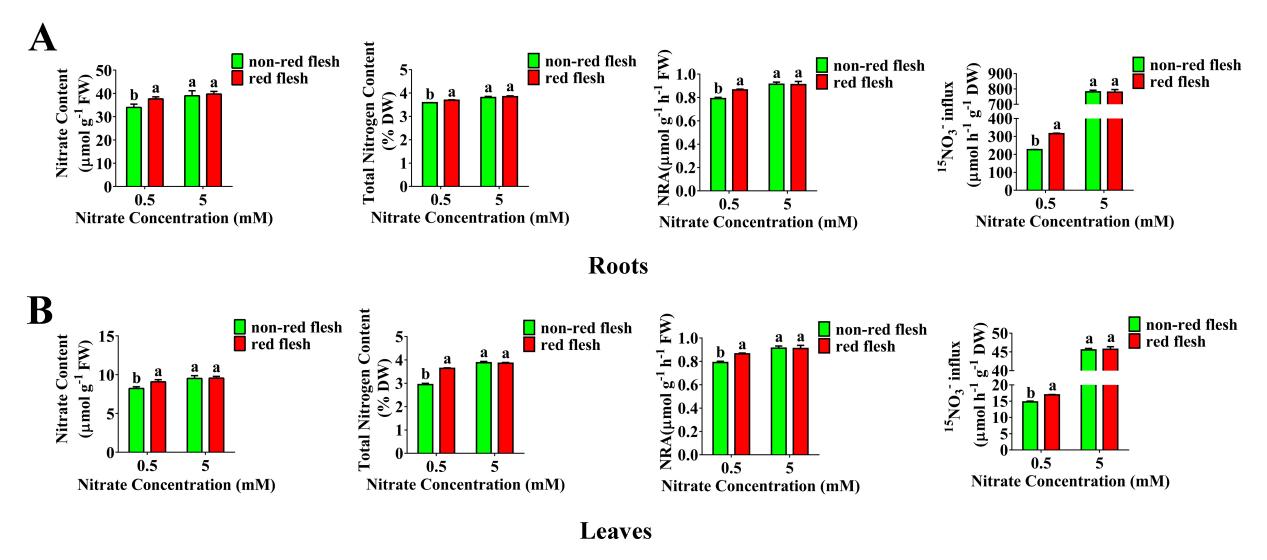


**Figure S2.** Detection of the nitrate content, total nitrogen content, NRA, and NDFF of the roots and leaves from red flesh and non-red flesh hybrid trees.

(A)-(B) Detection of the nitrate content, total nitrogen content, NRA, and NDFF(from left to right for each panel) of the roots (A) and leaves (B) from red flesh and non-red flesh hybrid trees. Data were mean±SD, and significant differences were determined using Tukey’s test, with different letters represent significant difference (a, b, c et al.).


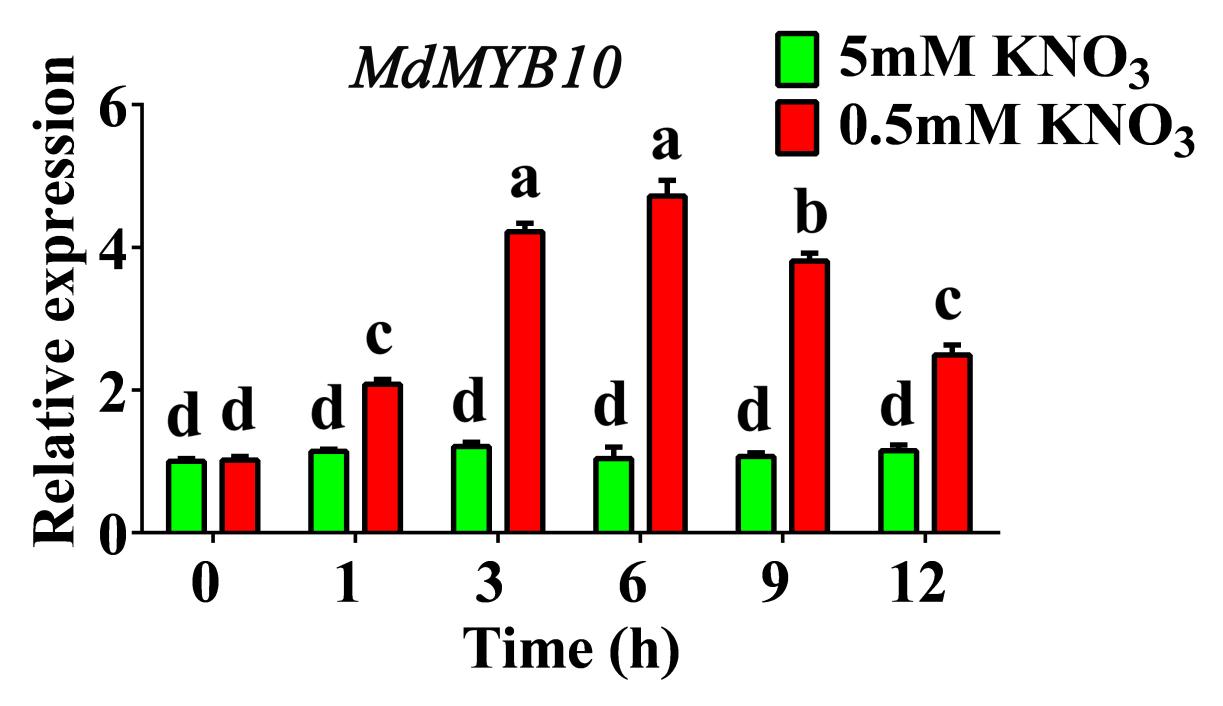


**Figure S3.** Transcript levels of *MdMYB10* in response to nitrate treatment.

Detection of the expression of *MdMYB10* in response to different nitrate condition (0.5 mM and 5 mM KNO3). Data were mean±SD, and significant differences were determined using Tukey’s test, with different letters represent significant difference (a, b, c et al.).


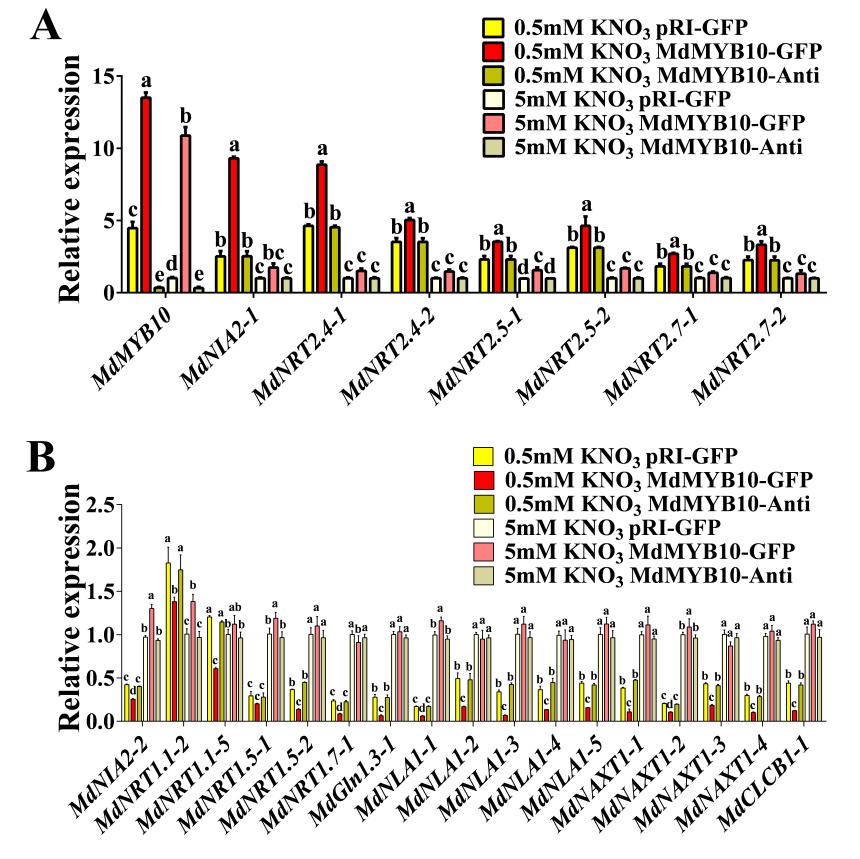


**Figure S4.** Transcript levels of nitrate-responsive genes under different nitrate treatments from *MdMYB10* transgenic calli.

1. –(B) Detect the transcripts of genes involved in nitrate uptake and transport (*MdNRTs*), nitrate assimilation (*MdNIA2s*), nitrogen metabolism (*MdGln1.3-1*), nitrate storage (*MdNAXT1s*, *MdCLCB1-1*) and nitrate signal (*MdNLA1s*) in the MdMYB10-OX and MdMYB10-anti transgenic calli in response to different nitrate treatments (0.5 mM and 5 mM KNO3). Data were mean±SD, and significant differences were determined using Tukey’s test, with different letters represent significant difference (a, b, c et al.).


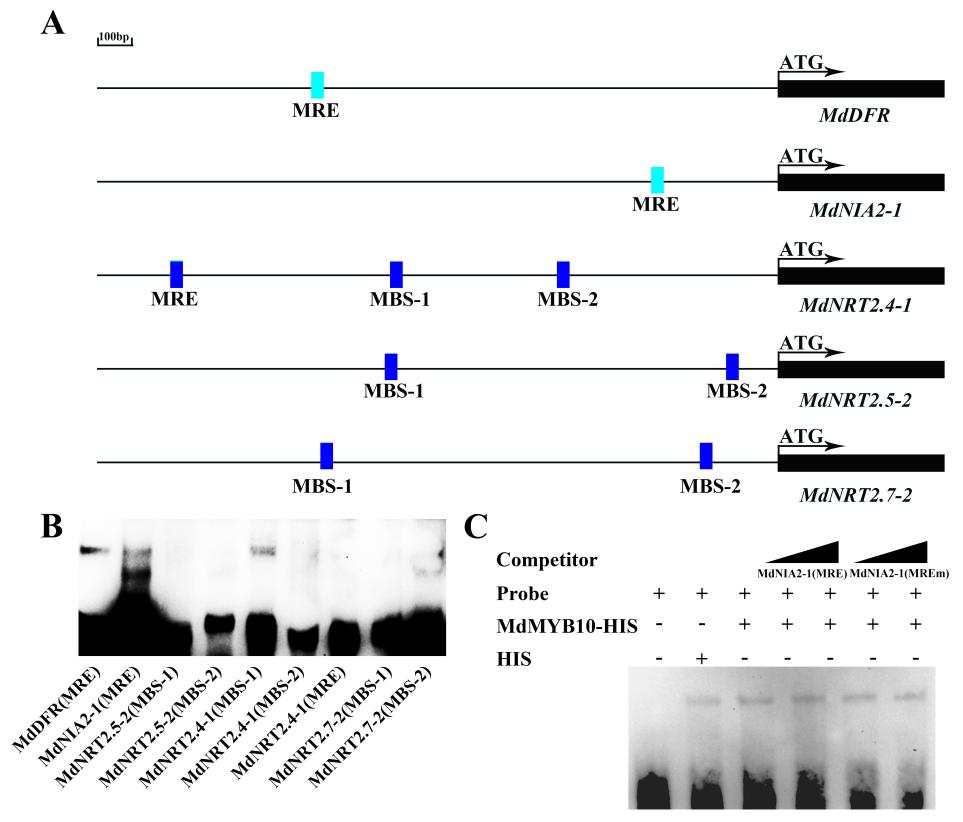


**Figure S5.** EMSA assay detects the binding of MdMYB10 to nitrate-responsive genes.

(A) Schematic diagram of the cis-element (MRE and MBS) in the promoter of *MdNIA2-1*, *MdNRT2.4-1*, *MdNRT2.5-2*, *MdNRT2.7-1*, with *MdDFR* as a positive control. Different colored boxes indicated the MRE and MBS *cis*-element in the promoter of each gene. (B) EMSA assay to detect the bind of MdMYB10 to the promoters of above genes *in vitro*. (C) Competitive interaction assay of MdMYB10 with *MdNIA2-1* promoter, competition was not observed when an unlabeled MRE competitor probe was used.


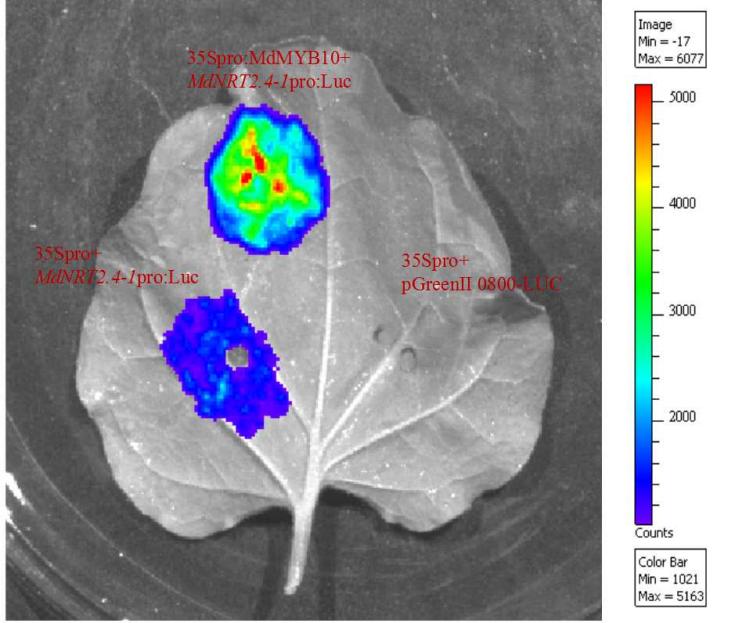


**Figure S6.** Transcriptional activation activity of MdMYB10 in regulating *MdNRT2.4-1*.

The recombinant plasmids of 35Spro:MdMYB10+MdNRT2.4-1pro:Luc, 35Spro+MdNRT2.4-1pro:Luc and 35Spro+pGreenII 0800-LUC empty vector were transformed into *N. benthamiana* leaves, and a charge-coupled device imaging apparatus was used to collect the LUC images.


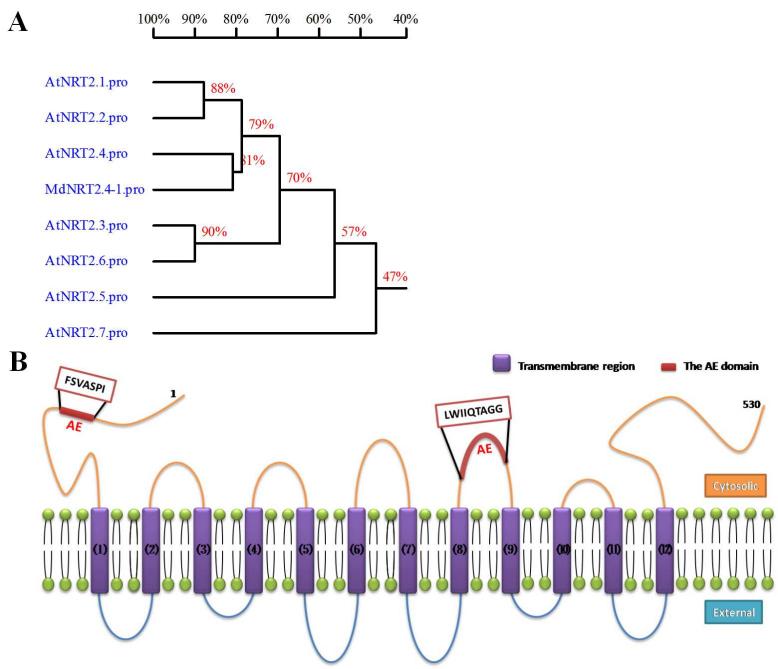


**Figure S7.** Phylogenetic tree and protein structure analysis of MdNRT2.4-1.

1. Phylogenetic tree of MdNRT2.-1 with *Arabidopsis* nitrate transporter genes AtNRT2s. (B) Prediction of protein structure of MdNRT2.4-1, which contained twelve transmembrane domain and two regulatory AE motifs on the cytosolic side.


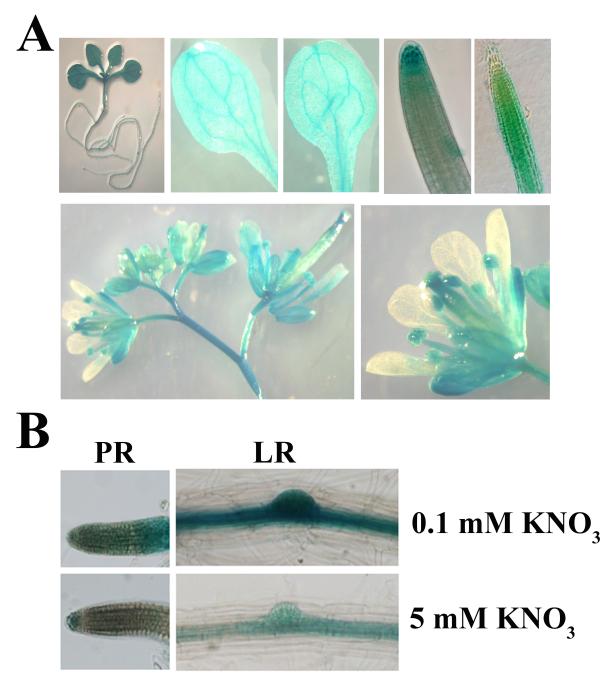


**Figure S8.** The temporal and spatial expression and nitrate response of *MdNRT2.4-1*.

1. The GUS staining of pMdNRT2.4-1::GUS reporter gene, the seeding, leaf, root and inflorescence were stained. (B) Histochemical staining of pMdNRT2.4-1::GUS transgenic *Arabidopsis* in response to nitrate. The pMdNRT2.4-1::GUS transgenic *Arabidopsis* were pre-treated on 1/2 MS medium for 7 d, then they were transferred to medium with different concentration of KNO (0.1 mM and 5 mM, with K^+^ up to the same concentration) for 6 h before staining.


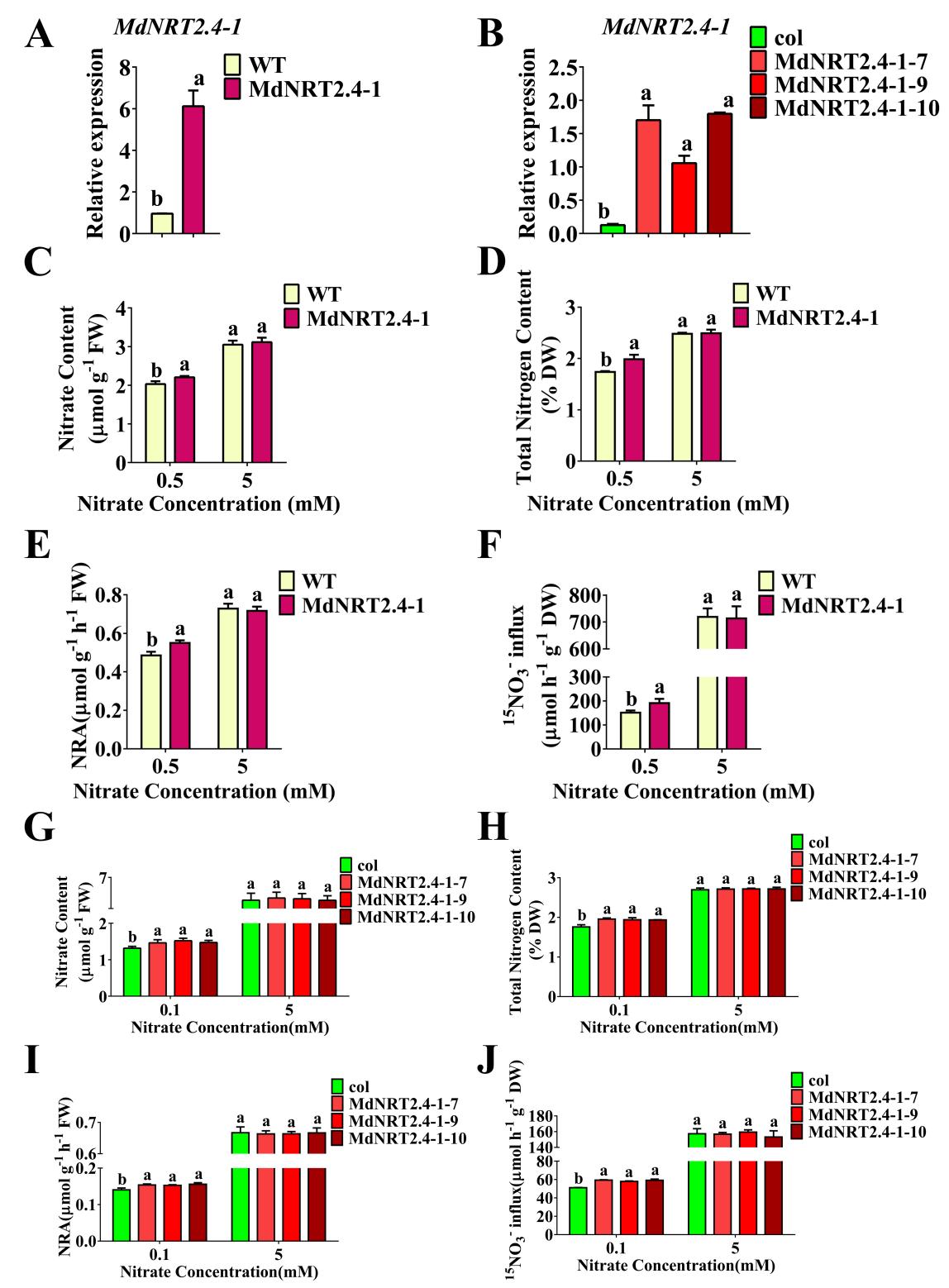


**Figure S9.** Identification of *MdNRT2.4-1* transgenic calli and *Arabidopsis* in regulating nitrate uptake and utilization.

1. -(B) Detection of transcript levels of *MdNRT2.4-1* in transgenic apple calli and *Arabidopsis*. (C)-(F) Detection of the nitrate content, NRA, total nitrogen content, and NDFF in the *MdNRT2.4-1* transgenic apple calli. (G)-(J) Detection of the nitrate content, NRA, total nitrogen content, and NDFF in the *MdNRT2.4-1* transgenic *Arabidopsis*. Data were mean±SD, and significant differences were determined using Tukey’s test, with different letters represent significant difference (a, b, c et al.).


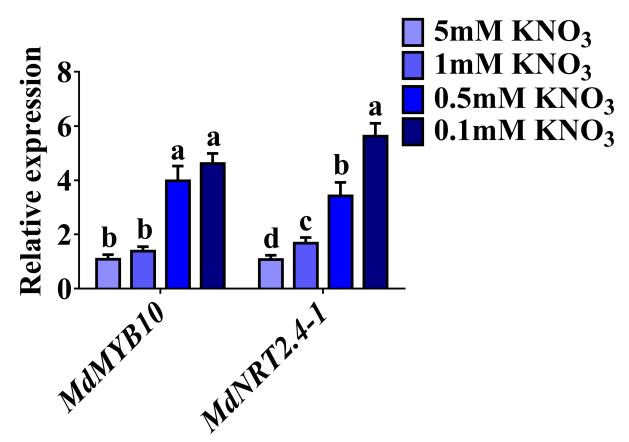


**Figure S10.** Transcript levels of *MdMYB10* and *MdNRT2.4-1* in response to different nitrate treatments.

Detection of the expression of *MdMYB10* and *MdNRT2.4-1* in response to different nitrate condition (0.1 mM to 5 mM KNO3). Data were mean±SD, and significant differences were determined using Tukey’s test, with different letters represent significant difference (a, b, c et al.).


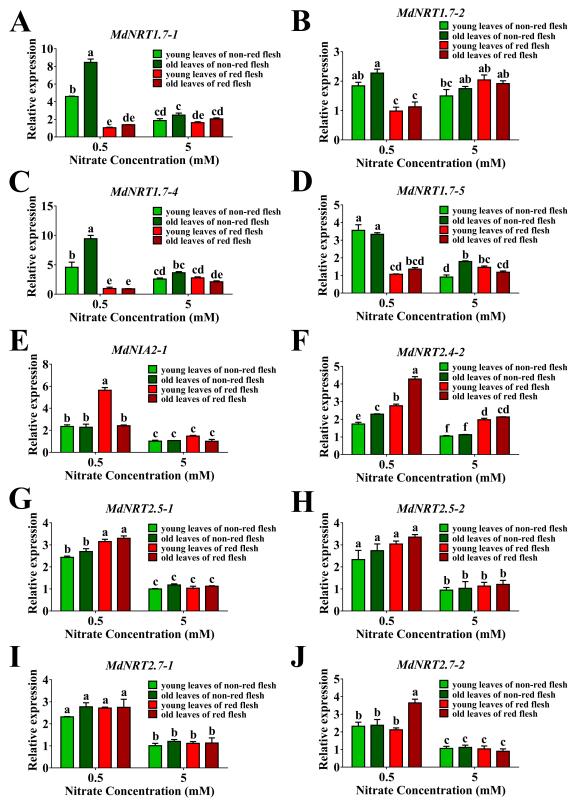


**Figure S11.** Expression pattern of nitrate-responsive genes in old and young leaves from red flesh and non-red flesh hybrid trees under different nitrate treatments.

(A)–(J) Detection of the expression pattern of nitrate-responsive genes *MdNRT1.7-1* (A), *MdNRT1.7-2* (B), *MdNRT1.7-4* (C), *MdNRT1.7-5* (D), *MdNIA2-1* (E), *MdNRT2.4-2* (F), *MdNRT2.5-1* (G), *MdNRT2.5-2* (H), *MdNRT2.7-1* (I), *MdNRT2.7-2* (J) in the old and young leaves from red flesh and non-red flesh hybrid trees under different nitrate treatments (0.5 mM and 5 mM KNO3). Data were mean±SD, and significant differences were determined using Tukey’s test, with different letters represent significant difference (a, b, c et al.).


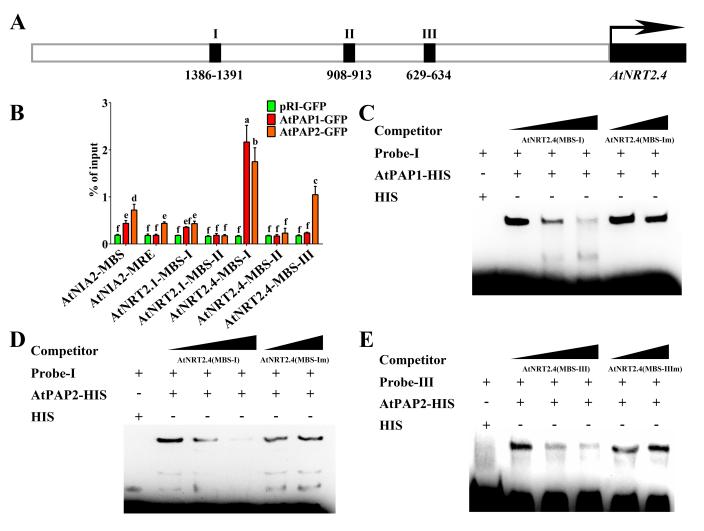


**Figure S12.** Detection of the binding of AtPAP1/2 to *AtNRT2.4* promoter.

1. Schematic diagram of the MBS *cis*-element in the promoter of *AtNRT2.4*, black colored boxes indicated the MBS *cis*-element. (B) *In vivo* ChIP-PCR assays detected the interaction of PAP1/2 with *AtNRT2.4* promoter using PAP1/2-GFP transgenic *Arabidopsis*. (C) EMSA assay detected the bind of PAP1 to the MBS-I *cis*-element of *AtNRT2.4* promoter. (C)-(D) EMSA assay detected the bind of PAP2 to the MBS-I (C) and MBS-III (D) *cis*-element of *AtNRT2.4* promoter.

**Table S1.** List of primers for gene cloning, vector construction, and qRT-PCR analysis.

| Primer name | Forward primer sequence (5' to 3') | Reverse primer sequence (5' to 3') |
| --- | --- | --- |
| 18S-RT | ACACGGGGAGGTAGTGACAA | CCTCCAATGGATCCTCGTTA |
| MdMYB10-RT | AGACCAATGTGATAAGACCTCAG | AACCAAAAACTTGTGAAGAGTTC |
| MdNAXT1-1-RT | TTTTAGCCACAGCCATCTCC | GACCCAATCAACGCAACAAT |
| MdNAXT1-2-RT | TTGTGTGGAGACCGATTTTG | TTTCAACCCGATTTCTGAGC |
| MdNAXT1-3-RT | TAATGTCAGTTGGGGGTTGG | TCACGAATGGGCTACCTTGT |
| MdNAXT1-4-RT | GAACTGCCAACGGACTTGAC | GGGGAAATACCATAAGCCAAA |
| MdNIA2-1-RT | CGGGAAGAAAGTCACACGAG | CCAGAAACACCAGCACCAGT |
| MdNIA2-2-RT | ACGACGAGAATGAGGACACC | GGCGGACCATAGACGAGTTA |
| MdNLA1-1-RT | TACTTGCCCTGACCATTGC | AGAAACCCACCACAGCAGAC |
| MdNLA1-2-RT | GGAGAATGATGCCGTTGATT | TCCAGGGTGAGATAGCCGTA |
| MdNLA1-3-RT | TACTTGCCCTGACCATTGC | AGAAACCCACCACAGCAGAC |
| MdNLA1-4-RT | TACTTGCCCTGACCATTGC | AGAAACCCACCACAGCAGAC |
| MdNLA1-5-RT | GCCTAAAGAAAAATGCCCTCT | CTCTACCCTCTCGGTCTGGA |
| MdNRT1.1-2-RT | AACCCTAACCGATGTTGAAGAA | TGCTTGGGACACTGAGAATG |
| MdNRT1.1-5-RT | GAGGCAATGGGGTTATGG | ACAACAAAGTCAAGTCCAACG |
| MdNRT1.5-1-RT | CTTCCTCTCTCTCCGCTCCT | CTCCCTCCGACAAAGTCAAG |
| MdNRT1.5-2-RT | GAAGAGCACAGCAAGGAAGG | AACCCAATAGCCCAAACTCC |
| MdNRT1.7-1-RT | GCGTCATCTCCTTTCTCACC | TCCCGATTCCACATCTTTG |
| MdNRT1.7-2-RT | GCAAAGTCCGAGCAACATTT | ATCCCACATTTAGTGAGCAACA |
| MdNRT1.7-4-RT | ACTCGGTCTCCTGCTACTGG | TTTCCTTCTTCGGTGGTTTG |
| MdNRT1.7-5-RT | CCTTGTTTTTAGCGGGGTCT | CCCTTTCGTCATTGTGAAGC |
| MdNRT2.4-1-RT | TCCTTCCAGCGTCCAAAGATGT | ATGGGAAGGTGTGGTGTTTGGT |
| MdNRT2.4-2-RT | CGAAGCAAGACATCGGAAAT | TAGCGTGGCCCTATCAAATC |
| MdNRT2.5-1-RT | TCGATCTGAAACTCCACACG | ACTTCTTCGCCACAACATCC |
| MdNRT2.5-2-RT | GCATTTCTCTGATGGGTGTG | CAGTTTCTGGTCCGCTTAGA |
| MdNRT2.7-1-RT | CGGGGTTGTTGTGCTTATTAC | CTCCCAGTGACCTTTTGGAC |
| MdNRT2.7-2-RT | GTCCAAAAGGCAAGCACCTA | CATCAGCACCGACAATCATC |
| MdGLN1.3-1-RT | CCAGGGGATGACAGTGAAGT | ATCTTGGCAGCGTTGAATCT |
| MdCLCB1-1-RT | CGAACACTTTCTTCCACTCCA | CAACAAGGCAGGCAAGGAT |
| AtPAP1-RT | TGGCACCAAGTTCCTGTAAG | AAGCCTATGAAGGCGAAGAA |
| AtPAP2-RT | TCCTTCATGCCTTGGACTCAA | CAAAGTGGCCCCATGTTCA |
| MdMYB10-GFP | GGGATCCATGGAGGGATATAACG | GGGATCCATTCTTCTTTTGAATGA |
| MdMYB10(Anti) | GAATTCTGCCTGGACTCGAGAGGAAGACA | GGATCCCCTGTTTCCCAAAAGCCTGTGAA |
| MdMYB10-HIS | CGGATCCCTAGCAGATAAGAGATGGAG | CGTCGACCTCTAGCTATTCTTCTTTTG |
| MdMYB10-pGADT7 | GGATCCAGATGGAGGGATATAACGAA | GAGCTCCTCTAGCTATTCTTCTTTTG |
| MdMYB10-pGBKT7 | CATATGATGGAGGGATATAACGAAAACCT | GTCGACCTCTAGCTATTCTTCTTTTGAA |
| MdNRT2.4-1-pCXSN | AATGGCAGATTCAGAAGGTG | CTAAACATGGGAAGGTGTGGT |
| MdNRT2.4-1_pro_-GUS | GGCTGCAGTCCCATTAACCAGT | ATTGTAATAGACCCCGAACCTG |
| MdNRT2.4-1-TRV2 | GAATTCTAAGCAGTTTCTGAGTTCCA | GAGCTCTGAAAACTTTGGCCTTGTG |
| MdNRT2.4-1_pro_(MBS-1)-pAbAi | CCCGGGGGCTGCAGTCCCATTAACCAGT | GTCGACATTGTAATAGACCCCGAACCTG |
| MdNRT2.4-1_pro_(MBS-1m)-pAbAi | CCCGGGGTCGACGAGTTGATTGTGTGAAG | GTCGACTCTAGATCGGATCGGATGAATTG |
| AtPAP1-GFP | GTCGACCTAATCAAATTTCACAGTCT | GTCGACCTAATCAAATTTCACAGTCT |
| AtPAP2-GFP | TCTAGAATGGAGGGTTCGTCCAAAGG | GGTACCATCAAGTTCAACAGTCTCTCC |
| AtPAP1-HIS | GGATCCATGGAGGGTTCGTCCAAAGG | GTCGACCTAATCAAATTTCACAGTCT |
| AtPAP2-HIS | GGATCCGAGGGTTCGTCCAAAGG | GTCGACCTAATCAAGTTCAACAGT |
| MdDRF(MRE)-ChIP | GGTTTATTTAGGGATCGTATTC | GAGAACATAGTCCAGCCCAAT |
| MdNIA2-1(MRE)-ChIP | CTCTTAAATAAGGCTAAGGATCTCG | TATGTCTGCCGCCAATCAAA |
| MdNRT2.4-1(MBS-1)-ChIP | GAGGGAAGATTCAGTT | ATACAAAATAGTGTTGTG |
| MdNRT2.4-1(MBS-2)-ChIP | CGTTCCCTGTGCTACTGTA | GAAAACAGAGCCAAATGAG |
| MdNRT2.4-1(MRE)-ChIP | GCAGTCCCATTAACCAGTCC | TTGATAGGGCCACGCTACG |
| MdNRT2.5-2(MBS-1)-ChIP | TTCTTCTCCACCTTCTCCA | GATGGCGATACGGGGAC |
| MdNRT2.5-2(MBS-2)-ChIP | ACAAGCAAGCAACTAACAGAC | TGTCACCAGCAGTTCCCATCA |
| MdNRT2.7-2(MBS-1)-ChIP | CAATGTTGTTGTGTCTTGTTTC | TCAGTCTCGACCTTGATTTC |
| MdNRT2.7-2(MBS-2)-ChIP | CTTCAATTCATTACATCGAACG | CAATGCAAGCAAACCTGTC |
| AtNIA(MBS)-ChIP | TCTGATAATATGTTTAATTGA | TTGATAATCCTTTTACCCCTG |
| AtNIA(MRE)-ChIP | TTCCAACATTCATACATAGGT | GATAAAATTAACAGATGTGAT |
| AtNRT2.1(MBS-Ⅰ)-ChIP | CGTTCCCTGTGCTACTGTA | GAAAACAGAGCCAAATGAG |
| AtNRT2.1(MBS-Ⅱ)-ChIP | CTAAGGCCACTCTAAAT | GAGGGTTATCTTTGTCATTGTA |
| AtNRT2.4(MBS-Ⅰ)-ChIP | TGTTGCACGTTTCCTC | TGTAATCAGATCCCTAA |
| AtNRT2.4(MBS-Ⅱ)-ChIP | CTTTAGGTAATGCTCTTTTTG | GATTCCTTTTCTTTCTAAG |
| AtNRT2.4(MBS-Ⅲ)-ChIP | CATGAGTATGGAGCTATA | CGTTAAACTTGGGTGAAG |
| MdDRF(MRE)-EMSA | TCTTCATTCACGAGATTCAAACCTAAAACTTCTGTTTTAGTGAA | TTCACTAAAACAGAAGTTTTAGGTTTGAATCTCGTGAATGAAGA |
| MdNIA2-1(MRE)-EMSA | AAGATTTCAGTTTAACAATTAGGTTGATTAGGAACCGGAACCAAA | TTTGGTTCCGGTTCCTAATCAACCTAATTGTTAAACTGAAATCTT |
| MdNIA2-1(MREm)-EMSA | AAGATTTCAGTTTAACAATTTCTAGGATTAGGAACCGGAACCAA | TTTGGTTCCGGTTCCTAATCCTAGAAATTGTTAAACTGAAATCTT |
| MdNRT2.4-1(MBS-1)-EMSA | TTATAGAGGGTTTTGAGTTGACCGTGTGAAGGGTTGTGAAGAGGC | GCCTCTTCACAACCCTTCACACGGTCAACTCAAAACCCTCTATAA |
| MdNRT2.4-1(MBS-1m)-EMSA | TTATAGAGGGTTTTGAGTTGATTGTGTGAAGGGTTGTGAAGAGGC | GCCTCTTCACAACCCTTCACACAATCAACTCAAAACCCTCTATAA |
| MdNRT2.4-1(MBS-2)-EMSA | ATGCTGCGTAGCATGGGCTACAACTGTACAAGCAATCATCCGCAT | ATGCGGATGATTGCTTGTACAGTTGTAGCCCATGCTACGCAGCAT |
| MdNRT2.4-1(MRE)-EMSA | AAGGGAAAAACCAATCATAAACCTAACTGCTATGTAACCCCCAGC | GCTGGGGGTTACATAGCAGTTAGGTTTATGATTGGTTTTTCCCTT |
| MdNRT2.5-2(MBS-1)-EMSA | ACCTCACCGACACAGACACCGGTCACGCAGGCACCGCTGCATTCC | GGAATGCAGCGGTGCCTGCGTGACCGGTGTCTGTGTCGGTGAGGT |
| MdNRT2.5-2(MBS-2)-EMSA | AAAATCAATCACGCTAAGATCAGTTGATAACGTACTTGAGTGGCC | GGCCACTCAAGTACGTTATCAACTGATCTTAGCGTGATTGATTTT |
| MdNRT2.7-2(MBS-1)-EMSA | TGAAATTAAGAAAACAAGAGCAGTTGCAACCTTCCCAAAAGAAAAA | TTTTTCTTTTGGGAAGGTTGCAACTGCTCTTGTTTTCTTAATTTCA |
| MdNRT2.7-2(MBS-2)-EMSA | TGTGTGGATAACCGAATATATAACTGTGATGAGTACAAGCAAAAGA | TCTTTTGCTTGTACTCATCACAGTTATATATTCGGTTATCCACACA |
| AtNRT2.4(MBS-Ⅰ)-EMSA | GGTTTTTATTTGTGAAGGTACAACTGTTATTGCTGTTACAATCTT | AAGATTGTAACAGCAATAACAGTTGTACCTTCACAAATAAAAACC |
| AtNRT2.4(MBS-Ⅰm)-EMSA | GGTTTTTATTTGTGAAGGTATGGTTGTTATTGCTGTTACAATCTT | AAGATTGTAACAGCAATAACAACCATACCTTCACAAATAAAAACC |
| AtNRT2.4(MBS-Ⅱ)-EMSA | AACATGATTTTTTTTTTCTGTAACTGTAAGACTATGTTATTTATT | AATAAATAACATAGTCTTACAGTTACAGAAAAAAAAAATCATGTT |
| AtNRT2.4(MBS-Ⅱm)-EMSA | AACATGATTTTTTTTTTCTGCGGTTGTAAGACTATGTTATTTATT | AATAAATAACATAGTCTTACAACCGCAGAAAAAAAAAATCATGTT |
| AtNRT2.4(MBS-Ⅲ)-EMSA | AGAATAGACTTGATAACTCGCAGTTGATAATGCCTCATGGTACAA | TTGTACCATGAGGCATTATCAACTGCGAGTTATCAAGTCTATTCT |
| AtNRT2.4(MBS-Ⅲm)-EMSA | AGAATAGACTTGATAACTCGACTGGTATAATGCCTCATGGTACAA | TTGTACCATGAGGCATTATACCAGTCGAGTTATCAAGTCTATTCT |

**Table S2.** Detection of the nitrate content, total nitrogen content, NRA, and NDFF of the roots and leaves from red flesh and non-red flesh hybrid trees at different growth stages.

| Hybridized combination | Date | Tissue | Cultivar | Nitrate(μmol·g-1 FW) | Total nitrogen(% DW) | NRA(μmol·g-1·h-1 FW) | NDFF % |
| --- | --- | --- | --- | --- | --- | --- | --- |
| Radiant × Orin | 4.16 | Roots | non-red flesh | 31.29±0.844 | 0.64±0.031 | 1.95±0.056 |  |
|  |  |  | red flesh | 35.78±0.978* | 0.83±0.032** | 2.16±0.038* |  |
|  |  | Leaves | non-red flesh | 6.71±0.196 | 2.07±0.030 | 0.38±0.012 |  |
|  |  |  | red flesh | 7.31±0.191* | 2.26±0.057* | 0.44±0.016* |  |
|  | 4.28 | Roots | non-red flesh | 31.08±0.562 | 0.52±0.006 | 3.09±0.047 | 3.57±0.028 |
|  |  |  | red flesh | 32.85±0.459* | 0.75±0.011** | 3.30±0.045* | 6.57±0.038*** |
|  |  | Leaves | non-red flesh | 6.72±0.062 | 2.35±0.006 | 0.68±0.005 | 0.59±0.021 |
|  |  |  | red flesh | 7.18±0.085* | 2.74±0.012** | 0.72±0.003* | 0.78±0.010* |
|  | 6.3 | Roots | non-red flesh | 21.01±0.022 | 0.41±0.003 | 1.93±0.012 | 2.13±0.037 |
|  |  |  | red flesh | 21.98±0.139* | 0.52±0.001* | 2.03±0.015* | 3.23±0.010** |
|  |  | Leaves | non-red flesh | 5.05±0.108 | 2.05±0.007 | 0.42±0.001 | 1.35±0.031 |
|  |  |  | red flesh | 5.39±0.035* | 2.26±0.023** | 0.45±0.006* | 3.23±0.010** |
|  | 7.4 | Roots | non-red flesh | 29.59±0.511 | 0.83±0.028 | 2.91±0.029 | 1.15±0.073 |
|  |  |  | red flesh | 32.16±0.661* | 0.93±0.011* | 3.08±0.061* | 1.51±0.075* |
|  |  | Leaves | non-red flesh | 7.16±0.143 | 2.43±0.019 | 0.72±0.031 | 4.61±0.073 |
|  |  |  | red flesh | 7.96±0.286* | 2.90±0.021*** | 0.80±0.016* | 5.35±0.108** |
|  | 8.3 | Roots | non-red flesh | 28.40±0.365 | 0.93±0.010 | 2.98±0.099 | 0.76±0.054 |
|  |  |  | red flesh | 30.34±0.527* | 1.46±0.037** | 3.52±0.027* | 1.03±0.079* |
|  |  | Leaves | non-red flesh | 6.47±0.070 | 2.53±0.042 | 0.62±0.015 | 4.17±0.131 |
|  |  |  | red flesh | 6.90±0.096* | 2.91±0.023** | 0.67±0.011* | 5.25±0.217** |
|  | 9.24 | Roots | non-red flesh | 48.42±1.587 | 1.45±0.023 | 5.19±0.160 | 0.72±0.010 |
|  |  |  | red flesh | 53.42±1.013* | 1.93±0.005** | 5.75±0.134* | 1.00±0.011* |
|  |  | Leaves | non-red flesh | 11.03±0.216 | 1.24±0.045 | 1.03±0.016 | 2.26±0.049 |
|  |  |  | red flesh | 11.69±0.187* | 1.68±0.016** | 1.12±0.027* | 2.66±0.037* |
| B9 × Royal Gala | 4.11 | Roots | non-red flesh | 30.12±0.650 | 0.74±0.034 | 2.91±0.018 |  |
|  |  |  | red flesh | 33.36±0.906* | 0.93±0.041* | 3.10±0.024* |  |
|  |  | Leaves | non-red flesh | 7.33±0.167 | 2.05±0.041 | 0.66±0.018 |  |
|  |  |  | red flesh | 8.14±0.194* | 2.23±0.056* | 0.73±0.020* |  |
|  | 4.25 | Roots | non-red flesh | 21.87±0.642 | 0.79±0.002 | 1.83±0.029 | 3.02±0.057 |
|  |  |  | red flesh | 23.83±0.651* | 1.04±0.002** | 1.95±0.016* | 6.03±0.038*** |
|  |  | Leaves | non-red flesh | 6.73±0.097 | 2.36±0.033 | 0.72±0.028 | 0.37±0.003 |
|  |  |  | red flesh | 7.39±0.172* | 2.83±0.036** | 0.78±0.011* | 0.55±0.008* |
|  | 6.3 | Roots | non-red flesh | 23.37±0.025 | 0.57±0.001 | 2.16±0.004 | 1.95±0.046 |
|  |  |  | red flesh | 25.35±0.039* | 0.80±0.003** | 2.25±0.007* | 2.92±0.021** |
|  |  | Leaves | non-red flesh | 5.61±0.120 | 2.20±0.005 | 0.47±0.001 | 1.67±0.053 |
|  |  |  | red flesh | 5.93±0.085* | 2.31±0.005* | 0.49±0.008* | 2.30±0.027** |
|  | 7.4 | Roots | non-red flesh | 42.19±0.822 | 0.77±0.023 | 3.74±0.050 | 1.18±0.080 |
|  |  |  | red flesh | 45.62±0.724* | 0.97±0.039* | 4.09±0.091* | 1.83±0.065* |
|  |  | Leaves | non-red flesh | 8.83±0.209 | 2.77±0.044 | 0.82±0.012 | 4.19±0.093 |
|  |  |  | red flesh | 9.55±0.181* | 3.08±0.066* | 0.88±0.013* | 4.75±0.217* |
|  | 8.3 | Roots | non-red flesh | 34.21±0.725 | 0.83±0.003 | 3.42±0.082 | 0.64±0.029 |
|  |  |  | red flesh | 38.37±0.748* | 1.43±0.021*** | 3.89±0.085* | 1.13±0.053* |
|  |  | Leaves | non-red flesh | 7.85±0.133 | 2.47±0.005 | 0.76±0.013 | 5.14±0.156 |
|  |  |  | red flesh | 8.62±0.113* | 2.84±0.016** | 0.81±0.019* | 5.79±0.192* |
|  | 9.24 | Roots | non-red flesh | 47.87±1.362 | 1.59±0.005 | 4.52±0.116 | 0.72±0.010 |
|  |  |  | red flesh | 52.08±0.901* | 2.16±0.026*** | 5.06±0.090* | 0.92±0.014* |
|  |  | Leaves | non-red flesh | 11.01±0.244 | 1.47±0.003 | 1.05±0.028 | 3.26±0.015 |
|  |  |  | red flesh | 11.74±0.224* | 1.88±0.036** | 1.16±0.028* | 3.66±0.029* |

**Table S3.** List of genes related to nitrate uptake and utilization that were differentially regulated in red flesh apple.

| GeneID | Log2 Ratio (Red/Green) | Up-Down Regulation (Red/Green) | P-value | Gene annotation |
| --- | --- | --- | --- | --- |
| MDP0000127691 | 12.91787 | Up | 1.37E-06 | MYB1 |
| MDP0000724757 | 11.04895 | Up | 0.0050815 | nitrate transporter 2.5 |
| MDP0000201530 | 3.676757 | Up | 2.53E-79 | nitrate transporter 2.7 |
| MDP0000148564 | 3.253788 | Up | 4.67E-67 | nitrate reductase 2 |
| MDP0000131368 | 2.721922 | Up | 5.68E-18 | nitrate transporter 2.7 |
| MDP0000239537 | 2.373565 | Up | 0.0038467 | nitrate transporter 2.4 |
| MDP0000722117 | 0.900987 | Up | 8.92E-54 | nitrite reductase 1 |
| MDP0000158753 | 0.585576 | Up | 7.79E-03 | nitrate transporter 1.5 |
| MDP0000442206 | 0.112002 | Up | 0.413354 | NADH-dependent glutamate synthase 1 |
| MDP0000183286 | 0.040529 | Up | 7.35E-01 | nitrate reductase 2 |
| MDP0000318858 | 0.021707 | Up | 0.713826 | NADH-dependent glutamate synthase 1 |
| MDP0000138927 | -0.07936 | Down | 0.417934 | nitrate transporter 1.5 |
| MDP0000408883 | -0.11186 | Down | 0.836678 | nitrate transporter 1.7 |
| MDP0000214328 | -0.5269 | Down | 0.547852 | nitrate transporter 1.1 |
| MDP0000266497 | -0.84494 | Down | 7.96E-148 | nitrate transporter 2.5 |
| MDP0000896511 | -0.86121 | Down | 0.0001445 | nitrate transporter 1.5 |
| MDP0000201061 | -1.01875 | Down | 0.0387452 | nitrate transmembrane transporters |
| MDP0000681768 | -1.10687 | Down | 4.33E-118 | nitrate transporter 1.7 |
| MDP0000585462 | -1.27343 | Down | 1.44E-79 | nitrate reductase 2 |
| MDP0000516531 | -1.61436 | Down | 0.0008945 | nitrate reductase 1 |
| MDP0000260604 | -2.24642 | Down | 6.81E-33 | nitrate transporter 1.5 |
| MDP0000280001 | -2.30475 | Down | 0 | nitrate reductase 2 |
| MDP0000151581 | -4.05828 | Down | 2.98E-39 | glutamine synthetase 1.3 |
| MDP0000321292 | -5.34712 | Down | 5.54E-01 | nitrate transporter 1.1 |
| MDP0000233661 | -6.85163 | Down | 0.553714 | nitrate transporter 1.7 |
| MDP0000214329 | -9.37813 | Down | 0.0424422 | nitrate transporter 1.1 |
| MDP0000548204 | -10.687 | Down | 0.022332 | nitrate transporter 1.1 |
| MDP0000173569 | -10.7394 | Down | 0.0001312 | nitrate transporter 1.5 |
